# Identification of an uncharacterized protein as a novel regulator of *Giardia lamblia virus* (GLV) infection in *Giardia duodenalis*

**DOI:** 10.1128/jvi.00883-25

**Published:** 2025-09-18

**Authors:** Zhiteng Zhao, Lili Cao, Jianqi Yuan, Shaoxiong Liu, Min Sun, Xin Li, Xiaocen Wang, Nan Zhang, Jianhua Li, Xichen Zhang, Pengtao Gong

**Affiliations:** 1 State Key Laboratory for Diagnosis and Treatment of Severe Zoonotic Infectious Diseases, Key Laboratory for Zoonosis Research of the Ministry of Education, Institute of Zoonosis, and College of Veterinary Medicine, Jilin University 623721, Changchun, China; 2 Jilin Academy of Animal Husbandry and Veterinary Medicine, Changchun, China; Wageningen University & Research, Wageningen, Netherlands

**Keywords:** GLV, capsid protein, regulatory factor, glycosyltransferase-stabilizing protein

## Abstract

*Giardia duodenalis* (*G. duodenalis*) is a prevalent intestinal protozoan parasite responsible for causing diarrhea, particularly in children and travelers. Although many infections are asymptomatic, they can result in severe malnutrition and cognitive developmental impairments in pediatric populations. *Giardia lamblia virus* (GLV) is a protozoan virus that specifically infects *G. duodenalis*. This virus is characterized by its non-segmented, non-enveloped double-stranded RNA structure and contains essential proteins such as RNA-dependent RNA polymerase (RdRp) and capsid protein (GLVCP). While the interactions between RdRp and host proteins have been extensively investigated, the function of GLVCP remains largely unexplored. In this study, we employed various methodologies, including mass spectrometry, co-immunoprecipitation (Co-IP), and bimolecular fluorescence complementation (BiFC), to identify and characterize the interactions between GLVCP and host proteins of *G. duodenalis*. Furthermore, we validated these interactions using CRISPR/dCas9 gene editing and protein overexpression techniques. Our research revealed that an uncharacterized protein (UCP) containing a glycosyltransferase-stabilizing (Gtf2) domain interacts with GLVCP. Under GLV influence, UCP modulates GlcNAc-mediated O-glycosylation and regulates multiple proteins that are involved in different pathways, such as ABC transporters, Malate Dehydrogenase (MDH), and cathepsin B (CTSB), creating a favorable intracellular environment to viral survival. Our findings provide crucial insights into the GLV life cycle and underscore the intricate interactions between the parasite and the virus. These results could inform the development of novel strategies for controlling *Giardia* infections and their associated diseases.

**IMPORTANCE:**

Our research has elucidated novel regulatory mechanisms between GLV and *G. duodenalis*, highlighting the complex interactions involving GLVCP and a host protein (UCP, a characterized protein containing a Gtf2 domain). This interaction enhances host GlcNAc-mediated O-glycosylation, inhibits ATP transport, and promotes the cleavage of GLVCP by cathepsin B, thereby facilitating the uncoating and release of the viral genome. These findings deepen our understanding of the GLV life cycle and fill gaps in our knowledge of the intricate dynamics between protozoan viruses and their hosts, providing valuable insights for the development of innovative strategies to control *Giardia* infections.

## INTRODUCTION

*Giardia* is a unicellular, flagellated, and binucleated intestinal protozoan capable of infecting nearly all vertebrates (1). It ranks among the primary pathogens responsible for diarrhea, particularly affecting children and travelers. In the majority of human cases, *Giardia* infection remains asymptomatic and latent, frequently going undetected. The infection is initiated when humans or livestock consume water or food contaminated with infectious cysts (2). Upon ingestion, these cysts encounter gastric acid and bile in the digestive tract, triggering their transformation into trophozoites within the proximal small intestine (3). The trophozoites subsequently colonize the intestinal wall using a specialized ventral sucker. Following successful colonization, the trophozoites proliferate rapidly, disrupting normal nutrient absorption in the small intestine and resulting in symptoms such as diarrhea and abdominal pain. In pediatric cases, *Giardia* infection can lead to significant malnutrition and cognitive developmental issues, with severe instances potentially culminating in chronic complications such as cognitive decline and growth retardation (1). Molecular analyses have categorized *Giardia* into eight distinct assemblages (Assemblages A–H) (4), with Assemblages A and B recognized as zoonotic, underscoring the parasite’s capability to infect both humans and animals (5).

*Giardia* possesses a streamlined genome, rendering it an essential model organism for exploring the origins of life and the mechanisms of gene regulation. Investigating the intricate interactions between *Giardia* and its host, as well as the interactions between viruses and *Giardia*, provides valuable insights into parasitic mechanisms and evolutionary dynamics (3). Viruses that specifically infect parasitic protozoa are referred to as parasitic protozoan viruses (PPVs). In recent years, virus-like particles (VLPs) have been detected in a range of parasitic protozoa, such as *Entamoeba histolytica* (6), *Leishmania hertigi* (7), *Cryptosporidium* (8), and *Trichomonas vaginalis* (9). These viral entities are characterized by several distinct features: they are non-enveloped and non-segmented, possessing an icosahedral morphology. Their genomic size ranges from approximately 4 to 7 kb, and they exhibit a diameter of 30–40 nm (10). Furthermore, these viruses are capable of encoding at least two proteins, namely, CP and RdRp. Initially, protozoan viruses were categorized within the single-stranded RNA virus family. However, this classification has been contested following the identification of additional viruses. For example, the *Entamoeba histolytica* virus (11), possessing a negative-sense single-stranded RNA (ssRNA) genome, has been reclassified under the Rhabdoviridae family, while the *Acanthamoeba polyphaga* mimivirus (APMV) (12), which contains double-stranded DNA (dsDNA), remains unclassified.

Investigations into the effects of PPVs on both the parasites themselves and the host-parasite interactions have revealed a variety of mechanisms. On one hand, PPVs can modulate the biological characteristics of the parasites, affecting their proliferation rates and metabolic pathways, such as gluconeogenesis and lipid metabolism (13). Conversely, PPVs can also alter the interactions between the parasite and the host, thereby modifying infection patterns and pathogenic mechanisms (14, 15). Moreover, PPVs may impair or delay the host immune system’s ability to recognize and eliminate the parasite, effectively serving as an “ally” to the parasite (16). Additionally, PPVs can exploit the parasite as a protective vehicle, shielding themselves from adverse environmental conditions (12, 17). These findings indicate that PPVs may play a crucial role in the survival strategies of parasites.

GLV is a specialized parasitic protozoan virus that exclusively infects *G. duodenalis* (18). In 1990, the International Committee on Taxonomy of Viruses (ICTV) officially classified it as a protozoan virus (19). GLV is taxonomically situated within the Riboviria realm, Orthornavirae subrealm, Duplornaviricota phylum, Chrymoviricetes class, Ghabrivirales order, Totiviridae family, and *Giardiavirus* genus. As a member of the Totiviridae family, GLV is characterized as a non-segmented, non-enveloped dsRNA virus (20). It possesses a linear, terminally open dsRNA genome of approximately 6.3 kb, a capsid protein of about 100 kDa, and a RdRp of around 190 kDa (18). The RdRp is critical in dsRNA viruses, facilitating viral genome replication, transcription, and RNA recombination (21). Previous research conducted in our laboratory has demonstrated that RdRp interacts with the chaperone protein DnaJ (GL50803_0017483) and Rab2a (GL50803_0015567), respectively. Inhibition of the transcription of these proteins results in a significant reduction in the copy number of GLV. The observed reduction cannot be solely attributed to the proliferation of *Giardia* trophozoites, suggesting that DnaJ (22) and Rab2a (unpublished data) play a positive regulatory role in GLV. However, the exact mechanisms by which this regulation occurs have yet to be fully elucidated. Additionally, as a critical protein of GLV, RdRp necessitates the coordinated involvement of multiple *Giardia* proteins to execute its biological functions. Numerous potential interacting partners of RdRp, identified in preliminary investigations, remain to be thoroughly explored (10).

In contrast to the extensively studied RdRp of GLV, GLVCP has been largely neglected. This oversight is partly attributable to the absence of a viral envelope and spikes on the GLV surface (23), features typically associated with viral entry and replication in other dsRNA viruses. GLV enters *Giardia* through non-caveolin-mediated endocytosis (24), with no clear host proteins identified to be involved in this process. Consequently, research on whether GLVCP is involved in GLV replication and affects its life cycle, as well as on the interaction between host proteins in *Giardia* and GLVCP in regulating viral proliferation, remains limited.

Recent advancements in gene-editing technologies, particularly the CRISPR/Cas system, have furnished researchers with robust tools for studying protein functions in *Giardia* (25). For example, CRISPR/Cas9 has been employed to knock out genes encoding proteins such as Myeloid Leukemia Factor (MLF) (26) and DNA Topoisomerase IIIβ (27), thereby revealing their roles in *Giardia* cyst formation. Despite the potential of CRISPR/Cas technologies, their application in elucidating the interactions between GLV and *Giardia* remains underexplored. This study seeks to identify and characterize the interactions between GLVCP and host proteins utilizing methodologies such as mass spectrometry, Co-IP, and BiFC. We employed CRISPR/dCas9 gene-editing and protein overexpression techniques to validate the functions of these interacting proteins and investigate their roles in GLV infection and replication. Through screening of potential interacting proteins, we have identified an uncharacterized protein capable of interacting with GLVCP, which contains a domain associated with Gtf2 protein function. Under the influence of GLV, this protein is capable of modulating O-glycosylation in *Giardia* trophozoites, mediating ATP transport, and facilitating the GLVCP cleavage and the viral genome release, thereby creating a favorable intracellular environment to GLV survival. This study is expected to provide critical insights into the GLV life cycle and enhance our understanding of parasite-virus interactions.

## MATERIALS AND METHODS

### *G. duodenalis* cultivation

GLV-free *G. duodenalis* isolates (Assemblage A, WB strain, ATCC No. 30957) and GLV-containing *G. duodenalis* isolates (designated as the VG strain, Assemblage A1, maintained in the parasitology laboratory of College of Veterinary Medicine, Jilin University) were cultured axenically at 37°C. The cultures were grown in modified TYI-S-33 medium, adjusted to a pH of 7.1, and supplemented with 12.5% fetal bovine serum (Every Green, Zhejiang) and 1 mg/mL bovine bile (Sigma-Aldrich, USA), until reaching the logarithmic growth phase.

### GLV isolation and construction of GLV-infected *Giardia*

GLV particles were isolated from the culture medium of VG strain trophozoites using the method described in reference 28. Briefly, the medium was collected, and unattached trophozoites and debris were removed by centrifugation at 2,000 × *g* for 10 min. The product underwent freeze-thaw cycles, grinding, and filtration through a 0.22 µm filter. It was then centrifuged at 15,000 × *g* for 30 min at 4°C. The viral pellet was ultracentrifuged at 100,000 *× g* for 2 h at 4°C. The virus was resuspended in cold PBS, and Cesium chloride (A620054, Sangon Biotech, China) was added to reach a density of 1.39 g/mL. Density gradient centrifugation was performed at 160,000 × *g* for 16 h. Virus-positive fractions, identified by nucleic acid electrophoresis, were dialyzed in 20% sterile PBS/glycerol, adjusted to a 50% glycerol, filter-sterilized, and stored at −80°C.

The concentration of intact GLV dsRNA was quantified based on previous studies, with 6 ng of viral RNA corresponding to 1 × 10^9^ virions (20). GLV-free *Giardia* WB strains were inoculated with 1,000 viral particles per trophozoite. Transmission stability was evaluated over 50 generations. Trophozoites were sub-cultured at 80%–90% confluence. Samples from the 1st, 2nd, 5th, 10th, 30th, and 50th generations, along with GLV-free and GLV-containing controls, were collected. Agarose gel electrophoresis was used to detect the GLV genome, and RNA and protein extractions were performed to evaluate GLV transcription and expression levels.

### Reagents, plasmids, and antibodies

G418 sulfate and Puromycin Dihydrochloride were purchased from YEASEN (Shanghai, China). Anti-HA magnetic beads were obtained from MCE (Shanghai, China). The pGEM-7zf (+) vector was synthesized by Shanghai Zeye Biotechnology Co., Ltd. The pcDNA3.1-N-3HA vector, pcDNA3.1-N-FLAG vector, pBiFC-bFosVC155 vector, pBiFC-bJunVN173 vector, pBiFC-VC155 vector, and pBiFC-VN173 vector were sourced from our laboratory collection. The antibodies utilized in this study included anti-GLVCP serum (1:2,000 for WB), anti-UCP serum (1:2,000 for WB), anti-CTSB serum, and anti-*Gl*tubulin serum (1:1,000 for WB), all of which were prepared in accordance with the polyclonal antibody preparation method described herein and stored in our laboratory. Additionally, the O-linked N-Acetylglucosamine Recombinant Rabbit mAb (S-R256) (rabbit mAb, 1:500 for WB, S0B0373, STARTER), the anti-HA Tag Recombinant Antibody (rabbit mAb, 1:5,000 for WB, 81290-1-RR, Proteintech), the anti-FLAG Tag antibody (rabbit mAb, 1:2,000 for WB, GB15939, Servicebio), and the multi-rAb Goat Anti-Rabbit Recombinant Secondary Antibody (H + L) (RGAR0001, 1:5,000 for WB, Proteintech) were acquired from their respective manufacturers.

### Plasmid construction

#### Target gene overexpression

Overexpression of the target gene was achieved by PCR amplification of coding sequences from *G. duodenalis* genomic DNA, with primers detailed in Table S1. The pGL-3HA-Neo vector was engineered by incorporating a 3 HA and Neo cassette into a BamHI-linearized pGEM-7zf (+) vector through Gibson assembly. Similarly, relative overexpression vectors were constructed by ligating the γ-giardin promoter and the target gene to the pGL-3HA-Neo vector via Gibson assembly.

#### Guide RNA design and cloning

Guide RNAs (~20 nucleotides) were designed for the CRISPR interference (CRISPRi) system using the EuPaGDT design tool (http://grna.ctegd.uga.edu/), specifically targeting the NGG protospacer adjacent motif (PAM) sequence. Potential off-target effects were evaluated against the *G. intestinalis* Assemblage A GiardiaDB-28 genome. The guide RNA oligonucleotides, listed in Table S2, contained four-base overhangs complementary to the vector sequence and were annealed by heating to 95°C for 5 min followed by gradual cooling to room temperature (RT) at a rate of 5°C per minute. Using Quick Ligase (M2200S, NEB), sticky-end fragments were subsequently cloned into a BbsI-digested dCas9g1pac vector, kindly provided by Prof. Scott Dawson from UC Davis, USA (25).

### Parasite transfection

The transfection of *Giardia* trophozoites was conducted as follows: A total of 10^7^ cells were resuspended and combined with purified cyclic vector, followed by incubation on ice for 10 min. Subsequently, the cells underwent electroporation in 4 mm cuvettes using a Bio-Rad Gene Pulser X cell, set at a voltage of 375 V, capacitance of 1,000 µF, and resistance of 750 Ω. After electroporation, the cells were transferred into 12 mL of medium after an additional 10 min incubation on ice. After incubation for a period of time in the absence of drugs, puromycin or G418 was introduced into the culture tubes at a final concentration of 15 µg/mL. The medium was replaced with fresh drug-containing medium every 2 days. Ultimately, the *Giardia* trophozoites were maintained under antibiotic selection with either puromycin or G418 for subsequent experimental procedures.

### RNA extraction and quantitative real-time PCR

Total RNA was extracted from freshly cultured *Giardia* trophozoites utilizing TransZol reagent (TransGen Biotech, Beijing) following the manufacturer’s protocol and subsequently resuspended in diethyl pyro-carbonate (DEPC)-treated water. RNA purity was evaluated using a Nanodrop 2000 spectrophotometer, with the 260/280 nm absorbance ratio serving as the assessment criterion. An aliquot of 2 µg of RNA was reverse transcribed into complementary DNA (cDNA) employing the All-In-One 5X RT MasterMix reagent (abm, Richmond, BC, V6V 2J5, Canada). Quantitative real-time PCR (qRT-PCR) was conducted using the Hieff qPCR SYBR Green Master Mix (YEASEN, China). The qRT-PCR conditions were as follows: an initial preincubation at 95°C for 5 min, followed by 40 cycles of 95°C for 10 s, and 60°C for 30 s. The sequences of the forward and reverse primers utilized for qRT-PCR are provided in Table S3. The transcripts of the *G. duodenalis* 18S ribosomal RNA gene (18S rRNA) and the actin gene were employed as reference genes to normalize mRNA levels in the samples. All experiments were performed with three independently prepared biological replicates.

### Western blot

Cells were collected and resuspended in a cell lysis buffer (Beyotime, China) containing a 1% protease inhibitor cocktail (Beyotime, China) for subsequent Western blot and immunoprecipitation analyses. Proteins extracted from various *Giardia* trophozoites and cell lines, including trophozoites with knock-down or overexpression plasmids and HEK293T cells containing BiFC or eukaryotic expression plasmids, were resolved using sodium dodecyl sulfate-polyacrylamide gel electrophoresis (SDS-PAGE) and subsequently transferred onto the polyvinylidene fluoride (PVDF) membrane (Millipore, Bedford, MA, USA). The membranes were blocked with Tris-buffered saline (TBS) containing 5% skimmed milk for 2 h at RT, followed by overnight incubation at 4°C with the appropriate primary antibodies in TBS supplemented with 0.05% Tween 20 (TBST). Thereafter, the membranes were treated with horseradish peroxidase-conjugated secondary antibodies and immunoreactive proteins were detected using an enhanced chemiluminescence system (Vigorous, Beijing, China). The intensities of the protein bands on the scanned film were analyzed for relative grayscale intensity using Image J software (NIH image software), with *Giardia lamblia* tubulin serving as the loading control.

### Protein-protein interaction assay

#### Co-IP

HEK293T cells were cultured in 6-well plates and transfected with either pcDNA3.1-N-3HA-GLVCP, pcDNA3.1-N-FLAG-UCP, or truncated fragments of pcDNA3.1-N-FLAG-UCP utilizing the Lipofectamine 2000 transfection reagent (Invitrogen). Whole-cell lysates (input) were harvested at 24 h post-transfection and subjected to centrifugation at 12,000 rpm for 5 min. The lysates were incubated overnight with HA-tag magnetic beads at a 1:50 ratio. Subsequently, the protein-bound beads were washed four times with TBST, each lasting 10 min. Following the washing steps, the protein-bound beads were analyzed via 8% SDS-PAGE and transferred onto PVDF membranes. The membranes were then blocked with 5% skimmed milk in TBS and incubated with specific primary antibodies overnight at 4°C. After washing with TBST, the membranes were treated with a goat anti-rabbit IgG secondary antibody. Finally, specific protein bands were detected using chemiluminescent methods.

#### BiFC

To conduct BiFC experiments, we designed primers to facilitate the linkage of BiFC experimental plasmids with GLVCP and UCP, respectively. The plasmids pBiFC-bFosVC155 and pBiFC-bJunVN173 were employed as positive controls. Using Gibson assembly, the *Fos* and *Jun* genes within these plasmids were substituted with the GLVCP and UCP genes to establish the experimental groups. Concurrently, the plasmids pBiFC-VC155 and pBiFC-VN173 functioned as negative controls. HEK293T cells were seeded onto slides in 6-well cell culture plates and subsequently transfected using Lipofectamine 2000 transfection reagent (Invitrogen). After a 24 h incubation period, fluorescence microscopy was utilized for detection.

#### Protein interaction prediction by AlphaFold 3

The three-dimensional crystal structure models of GLVCP and UCP were accurately predicted using the advanced AlphaFold 3 algorithm. Moreover, the complex structures formed the interaction of GLVCP with UCP were precisely determined through AlphaFold 3 and subsequently subjected to comprehensive analysis using the Schrödinger software suite (29).

### Transcriptome-metabolome correlation analysis

Morphologically intact *Giardia* VIG and WB strain trophozoites (approximately 5 × 10^7^ each) were collected from the culture medium. Pre-cooled PBS was added, and the cells were gently agitated for 1 min to facilitate washing. The PBS was then discarded, and this washing step was repeated twice to ensure complete removal of the culture medium. The trophozoites were subsequently subjected to an ice bath for 30 min and centrifuged at 2,000 *g* for 10 min.

For transcriptomic analysis, the pellets were resuspended in TransZol, precipitated, and stored at −80°C. These samples were then sent to Shanghai Bioprofile Technology Co., Ltd. for library construction and next-generation sequencing using the Illumina HiSeq platform.

For the preparation of metabolomics samples, the pellets were resuspended in a minimal volume of pre-cooled phosphate-buffered saline (PBS), rapidly frozen in liquid nitrogen for 1 min, and subsequently stored at −80°C. These samples were then dispatched to Shanghai Bioprofile Technology Co., Ltd. for ultra-high-performance liquid chromatography-tandem mass spectrometry (UHPLC-MS/MS) analysis.

### Electron microscopy

Detailed procedures for scanning electron microscopy (SEM) and transmission electron microscopy (TEM) are provided in the Supplementary Material.

### Statistical analysis and image generation

Data are expressed as means ± standard error from three independent samples. Statistical significance was assessed using a student’s *t*-test (paired or unpaired) or one-way or two-way analysis of variance (ANOVA), followed by Dunnett’s post hoc test for multiple comparisons. Significance levels are indicated as *P < 0.05, **P < 0.01, ***P < 0.001, ****P < 0.0001, with ns denoting not significant (*P* > 0.05). Figures were composed using Adobe Illustrator 2024, while schematic illustrations were created using Figdraw 2.0 within the HOME for Researchers platform.

## RESULTS

### GLV can enhance *Giardia* proliferation

**Fig 1:**
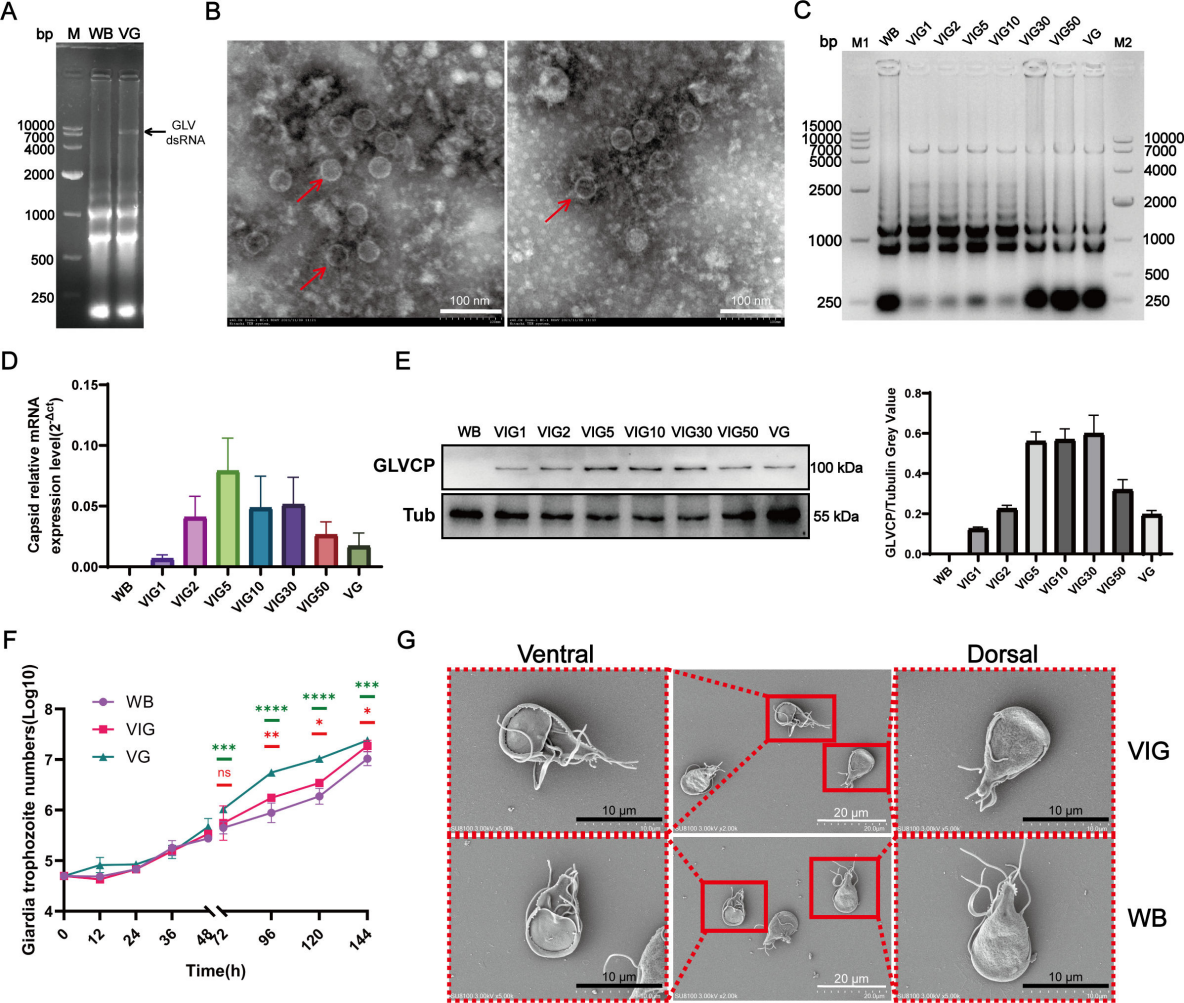
Construction of GLV-infected *G. duodenalis*. (**A**) Agarose gel electrophoresis analysis of total RNA from the WB strain and VG strain. Black arrow indicated the viral dsRNA in total RNA of VG strain. (**B**) Electron microscopy with negative staining of GLV isolated from the VG strain. The red arrow indicates the viral particles. Scale bar, 100 nm. (**C, D**) Agarose gel electrophoresis (**C**) and quantitative real-time PCR analysis (**D**) demonstrate the presence and consistent detection of the viral dsRNA in the total RNA of the VIG strain across different generations. M1, M2: DNA ladder; Lane 1: WB strain; Lanes 2–7: generations 1, 2, 5, 10, 30, and 50 of the VIG strain, respectively. Lane 8: VG strain. (**E**) GLVCP in different generations of the VIG strain were detected using Western blot and gray value analysis of GLVCP/*Gl*Tubulin. (**F**) Growth curves of *G. duodenalis* strains (WB, VIG, and VG) were monitored over a 7-day period. The VIG strain and VG strain exhibited distinct proliferation patterns compared to the WB strain. Data are represented as mean ± standard deviation (*n* = 3 independent experiments). A statistical analysis was conducted on the growth curves after 72 h. The green asterisks represent the statistical significance level between WB and VG strains, while the red asterisks indicate the statistical significance level between WB and VIG strains. (**G**) SEM observation of the morphology of *G. duodenalis* trophozoites from the VIG strain (top) and WB strain (bottom). SEM analysis revealed no significant morphological differences in the ventral (left) and dorsal (right) surfaces of the VIG strain compared to the WB strain. Scale bar, 10 µm and 20 µm.

As a member of the dsRNA virus family, the life cycle of GLV necessitates the involvement of various host proteins. To identify the *Giardia* proteins that facilitate the GLV life cycle through the GLVCP, we isolated and purified GLV from *Giardia* VG strains. This purified GLV was used to naturally infect WB strains, which were subsequently designated as VIG strains (Virus-infected *Giardia*). Agarose gel electrophoresis of total RNA revealed a distinct band corresponding to viral dsRNA in the VG strain (Fig. 1A), which was absent in the WB strain, confirming the presence of GLV in the VG strain. The GLV particles, purified via density gradient centrifugation from the VG strain, were stained with phosphotungstic acid, allowing for the observation of their morphology through transmission electron microscopy (Fig. 1B). The particles exhibited an approximately diameter of 38 nm.

To assess the growth stability of our engineered GLV-infected *Giardia*, we collected trophozoites from the VIG strain at the 1st, 2nd, 5th, 10th, 30th, and 50th generations (designated as VIG1, VIG2, VIG5, VIG10, VIG20, VIG30, and VIG50), along with trophozoites from the WB strain and the VG strain, for protein and total RNA analysis. Agarose gel electrophoresis confirmed the persistent presence of the GLV genome across successive generations of the VIG strain (Fig. 1C). The transcription and expression levels of GLV capsid proteins exhibited fluctuations during transmission. Notably, as the number of generations increased, the transcriptional and translational levels of GLV in the VIG strain gradually aligned with those observed in the VG strain, indicating an increasingly stable relationship between GLV and *Giardia* (Fig. 1D and E).

To determine the impact of GLV on the proliferation of *Giardia* trophozoites, we conducted a 7-day observational study. The findings indicated that the proliferation rate of GLV-infected *Giardia* (VG and VIG strains) exceeded that of WB strain (Fig. 1F). Scanning Electron Microscopy (SEM) demonstrated that the morphology of the VIG strain trophozoites remained predominantly unchanged (Fig. 1G). These observations suggest that GLV-infected *Giardia* can be stably transmitted to subsequent generations. Furthermore, while GLV enhances the proliferation of *Giardia*, it does not affect its morphological characteristics. Based on these results, we used the VIG strain (designated as VIG50) that stably harbors GLV for subsequent studies.

### Multiple functional proteins are involved in GLV replication

**Fig 2:**
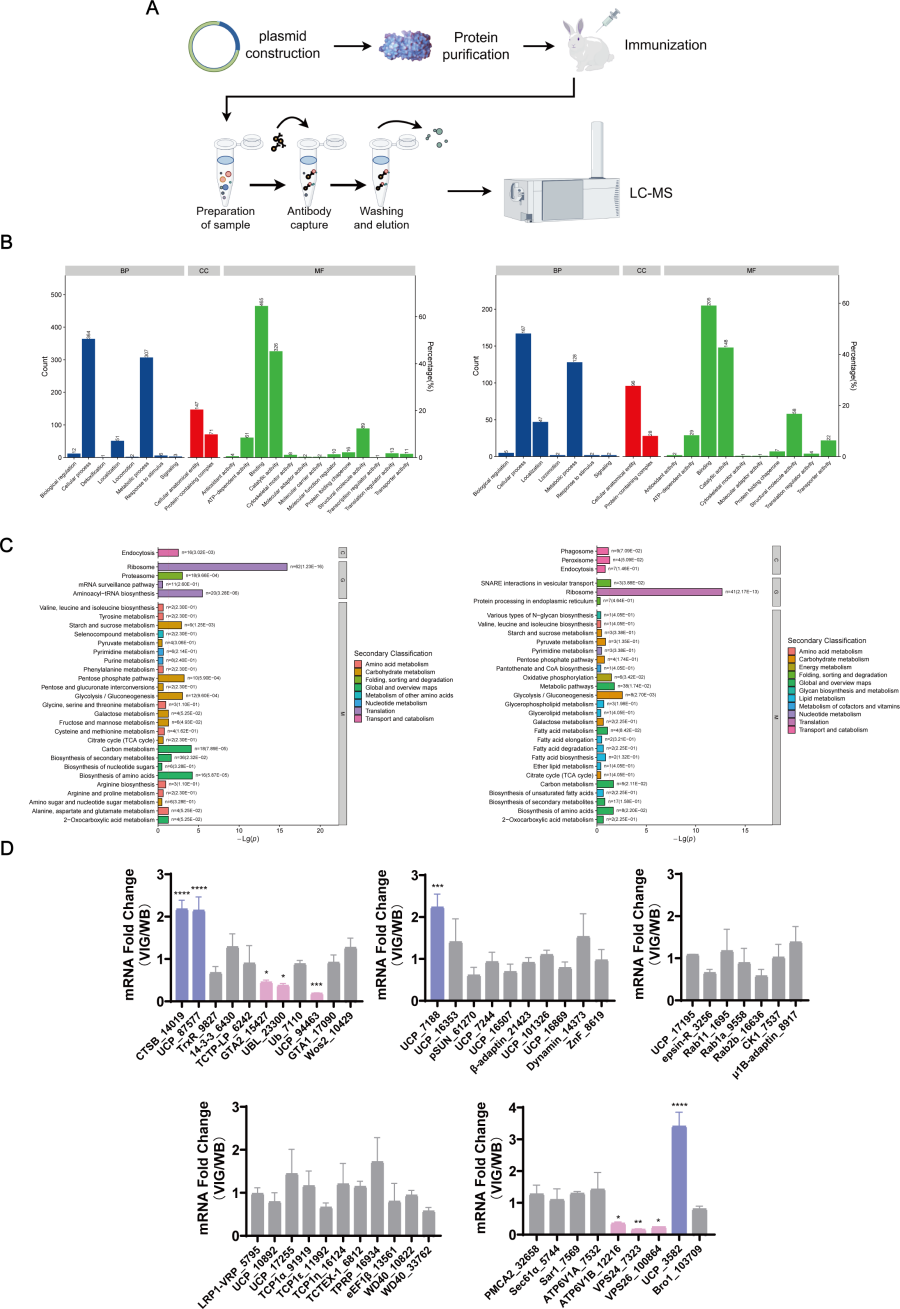
Screening for interacting proteins of the GLVCP. (**A**) Schematic illustration of the workflow for identifying potential interactors of the GLVCP, including plasmid construction, protein expression and purification, polyclonal antibody generation, immunoprecipitation, and LC-MS/MS analysis. (**B**) Gene Ontology (GO) analysis of cytosolic proteins (left) and membrane proteins (right) of *G. duodenalis*. (**C**) KEGG secondary classification analysis of cytosolic proteins (left) and membrane proteins (right) of *G. duodenalis*. (**D**) Differential transcription levels of potential GLV life cycle-related interacting proteins in VIG and WB strains analyzed by qRT-PCR. All proteins are shown using their generic abbreviations followed by the *Giardia lamblia* gene identifier, e.g., an uncharacterized protein with the identifier GL50803_0094463 is labeled UCP_94463. Results are expressed as mean ± standard deviation from three separate experiments, *P < 0.05, **P < 0.01, ***P < 0.001, ****P < 0.0001.

GLV has the capability to regulate the proliferation of *G. duodenalis*; however, there have been limited investigations into the mechanisms by which GLV utilizes *Giardia* proteins to modulate its own life cycle. GLVCP is identified as the initial component that interacts with *Giardia* proteins. Consequently, we developed a prokaryotic GLVCP expression vector, purified the capsid protein, and generated polyclonal antibodies (Fig. 2A). These polyclonal antibodies specifically detect GLV in the VIG strain. Subsequently, we performed immunoprecipitation of trophozoite proteins from the VIG strain using Anti-GLVCP polyclonal antibodies and rabbit-derived IgG, leading to the identification of 352 candidate proteins interacting with GLVCP via LC-MS/MS analysis (Table S4). To ensure the reliability of the identified interacting proteins, we extracted membrane and cytoplasmic proteins from *Giardia*, screening 1,018 cytoplasmic proteins and 563 membrane proteins, also through LC-MS/MS analysis (Table S5).

In conjunction with the Gene Ontology (GO) analysis (Fig. 2B) and Kyoto Encyclopedia of Genes and Genomes (KEGG) analysis (Fig. 2C) of these proteins, 48 proteins potentially implicated in the viral life cycle processes, such as endocytosis, adsorption, and protein synthesis, were identified from 352 candidate interactions of GLVCP. The qRT-PCR analysis of the mRNA levels of these 48 proteins in the VIG and WB strains (Fig. 2D) revealed that ten proteins, including cathepsin B (CTSB_14019), trophozoite antigen GTA-2 (GTA2_15427), ubiquitin family protein (UBL_23300), v-ATP synthase subunit B (ATP6V1B_12216), vps24 (VPS24_7323), vps26 (VPS26_100864), and four uncharacterized proteins (UCP_87577, UCP_94463, UCP_7188, and UCP_3582), exhibited significant alterations in transcript levels in response to GLV. These findings suggest that these proteins are likely involved in GLV replication within *Giardia* trophozoites.

### The novel UCP interacts with GLVCP

**Fig 3:**
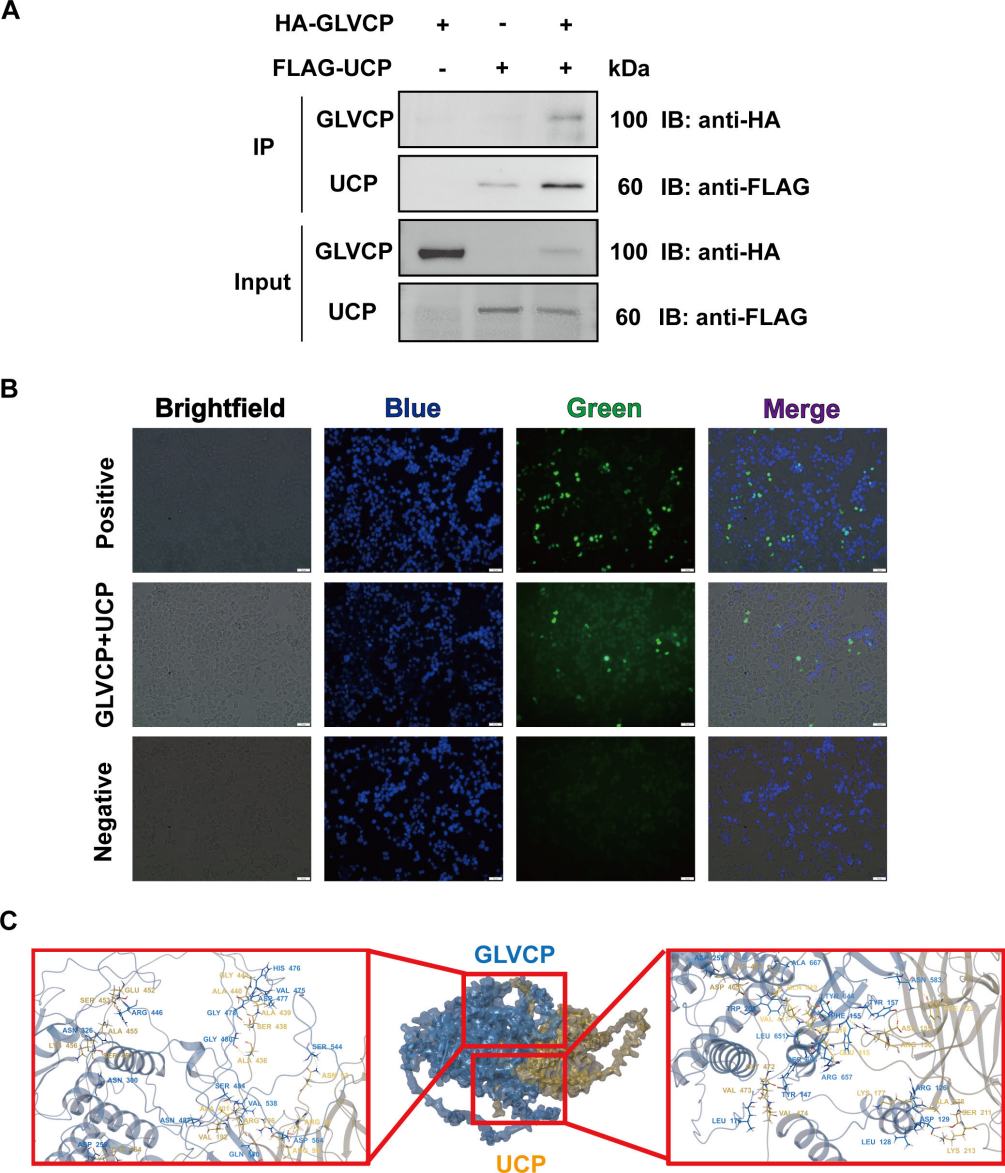
Interaction between UCP and GLVCP. (**A**) Western blot analysis of the interactions between GLVCP and UCP. 3HA-vector and FLAG-vector were used as controls. Input: Cell lysate before immunoprecipitation. IB, Immunoblotting. (**B**) Fluorescence identification of the relationship between GLVCP and UCP. Cell co-transfected with Fos-VN155-vector and Jun-VN173-vector were used as the positive controls and VN155-vector and VN173-vector were used as the negative controls, respectively. The nuclei stained by Hoechst33342 are shown in blue. Scale bar, 50 µm. (**C**) AlphaFold 3 prediction results of the key structural domains of GLVCP binding to UCP.

Based on the aforementioned results, it is evident that GLV infection influences the transcriptional levels of certain *Giardia* proteins, including CTSB_14019, UCP_87577, GTA2_15427, UBL_23300, UCP_94463, UCP_7188, ATP6V1B_12216, VPS24_7323, VPS26_100864, and UCP_3582. These proteins may be directly regulated by GLVCP. To ascertain whether there are trophozoite proteins that directly interact with GLVCP, we engineered recombinant eukaryotic expression vectors for these 10 proteins and validated their interactions with GLVCP using Co-IP. The Western blot analysis (Fig. 3A) revealed that an uncharacterized protein (GL50803_0094463, NCBI ID: XP_001708344.1), referred to as UCP, exhibited a direct interaction with GLVCP as demonstrated by Co-IP, corroborating the findings from the BiFC experiments (Fig. 3B). Furthermore, three-dimensional structural analysis and interaction site modeling of the GLVCP and UCP structures indicated a stable binding domain between UCP (depicted in yellow) and GLVCP (depicted in blue) (Fig. 3C). Notably, hydrogen bonds were observed between amino acid Asn93 of UCP and amino acid Ser544 of GLVCP, amino acid Arg156 of UCP and amino acid Phe155 of GLVCP, as well as amino acid Val192 of UCP and amino acid Asn487 of GLVCP. These interactions provide additional confirmation of the relationship between UCP and GLVCP. The 10 most significant interaction sites between UCP and GLVCP, as predicted by AlphaFold 3, are detailed in Table S6.

**Fig 4:**
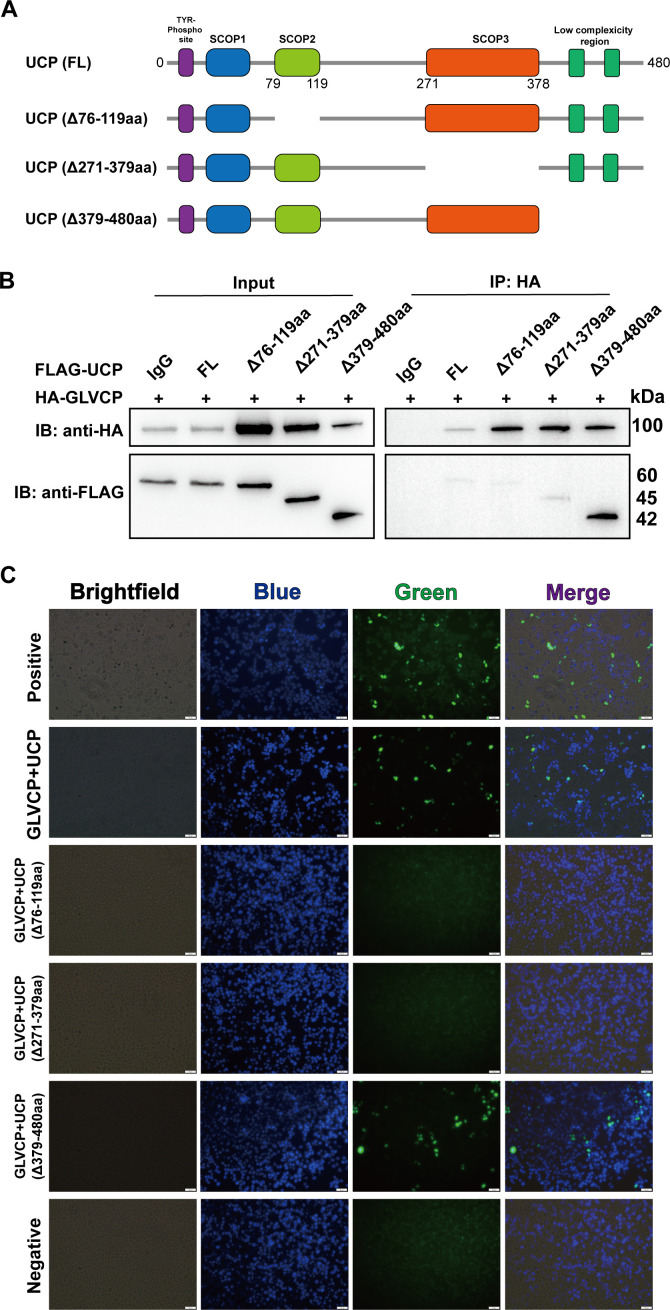
Interaction between UCP truncated fragments and GLVCP. (**A**) Schematic diagram of the UCP structural domains and three truncated fragments forms of UCP. (**B**) Western blot analysis of the interaction between GLVCP and UCP truncated fragments. Input: Cell lysate before immunoprecipitation. IB, Immunoblotting. (**C**) Fluorescence identification of the relationship between GLVCP and UCP truncated fragments. Cells co-transfected with Fos-VN155-vector and Jun-VN173-vector were used as the positive controls and VN155-vector and VN173-vector were used as the negative controls, respectively. The nuclei stained by Hoechst33342 are shown in blue. Scale bar, 50 µm.

To elucidate the principal domains facilitating UCP binding to GLVCP, we conducted a structural prediction analysis of the UCP amino acid sequence using InterPro (https://www.ebi.ac.uk/interpro/). This analysis revealed that UCP possesses an N-terminal tyrosine-phosphorylation site, three SCOP domains (SCOP1, SCOP2, and SCOP3), and a C-terminal characterized by low complexity (Fig. 4A). Guided by these predicted structural domains, we constructed truncated fragments of UCP utilizing Gibson assembly. Notably, protein expression assays indicated that truncation of the initial 69 amino acids at the N-terminal end of UCP resulted in a lack of protein expression (unpublished data), suggesting that this segment is crucial for UCP expression. In contrast, truncations encompassing amino acids 76–119, 271–379, or 379–480 did not impede the interaction with GLVCP (Fig. 4B). However, BiFC experiments demonstrated that only the UCP variant retaining the first 378 amino acids was capable of interacting with GLVCP within 293T cells (Fig. 4C). The WB results demonstrated a notably stronger interaction between the UCP truncated variant containing the first 378 amino acids and GLVCP compared to other variants, aligning with protein-protein interaction modeling findings. These results suggest that UCP directly interacts with GLVCP, with the initial 378 amino acids being crucial for this interaction.

### The novel UCP regulates the replication of GLV in *G. duodenalis*

Protein-protein interaction assays confirmed UCP’s direct interaction with GLVCP, implying its potential role in the GLV’s life cycle. To investigate UCP’s effect on GLV replication, we developed UCP overexpression and knockdown strains. The overexpression plasmid was created by inserting the UCP gene into the MCS region of a backbone plasmid, assembled using Gibson assembly with a γ-giardin promoter, 5′-UTR, and 3′-UTR (Fig. S1A). For knockdown, gRNA was annealed and ligated to a BbsI-digested dCas9g1pac plasmid, forming the UCP knockdown plasmid (Fig. S1B). To optimize knockdown efficiency, we synthesized the top three guide RNAs (gRNAs) with the highest scores as predicted by the gRNA design platform.

Variability in knockdown efficiency can arise from different gRNAs. Transient transfection experiments demonstrated that all three gRNAs achieved satisfactory knockdown efficiency (Fig. S2). Among them, gRNA2 was selected for further investigation (Fig. S2B). The analysis of qRT-PCR (Fig. S3A and B) and Western blot (Fig. S3C) showed that 6 h post-transient transfection with the UCP overexpression plasmid, both transcriptional and protein expression levels of UCP were elevated compared to other time points. Consequently, this time point was chosen for subsequent drug screening. Similarly, qRT-PCR (Fig. S3D and E) and Western blot analysis (Fig. S3F) identified that the transcriptional and protein expression peaks of the dCas9 protein occurred 12 h post-transient transfection. This time point was, thus, selected for the drug screening in the context of stable transfection in *G. duodenalis*. Following the determination of the optimal time points for drug addition, we established the appropriate drug concentrations and plasmid transfection dosages. The experimental results showed that a G418 concentration of 15 µg/mL demonstrated effective screening efficiency (Fig. S3F), whereas the optimal concentration for puromycin was determined to be 30 µg/mL (Fig. S4B). The optimal dosages for plasmid transfection were identified as 20 µg and 30 µg for the two plasmids, respectively (Fig. S4C and D).

Subsequent to the optimization and selection of UCP overexpression and knockdown strains, we evaluated the impact of the transfected plasmids on strain proliferation by monitoring trophozoite growth. Trophozoite enumeration revealed that the UCP knockdown strain exhibited the highest proliferation rate, in contrast to the wild-type trophozoites, which exhibited the lowest rate (Fig. S5A). To ascertain that the knockdown or overexpression of *Giardia* proteins was not influenced by the presence of GLV, we performed UCP knockdown and overexpression experiments in both WB and VIG strains. The qRT-PCR (Fig. S5B and C) and Western blot (Fig. S5D and E) analyses confirmed that the constructed knockdown and overexpression plasmids were effective in modulating UCP expression levels in the trophozoites of both VIG and WB strains. The presence of GLV did not affect the knockdown and overexpression of trophozoite proteins.

**Fig 5:**
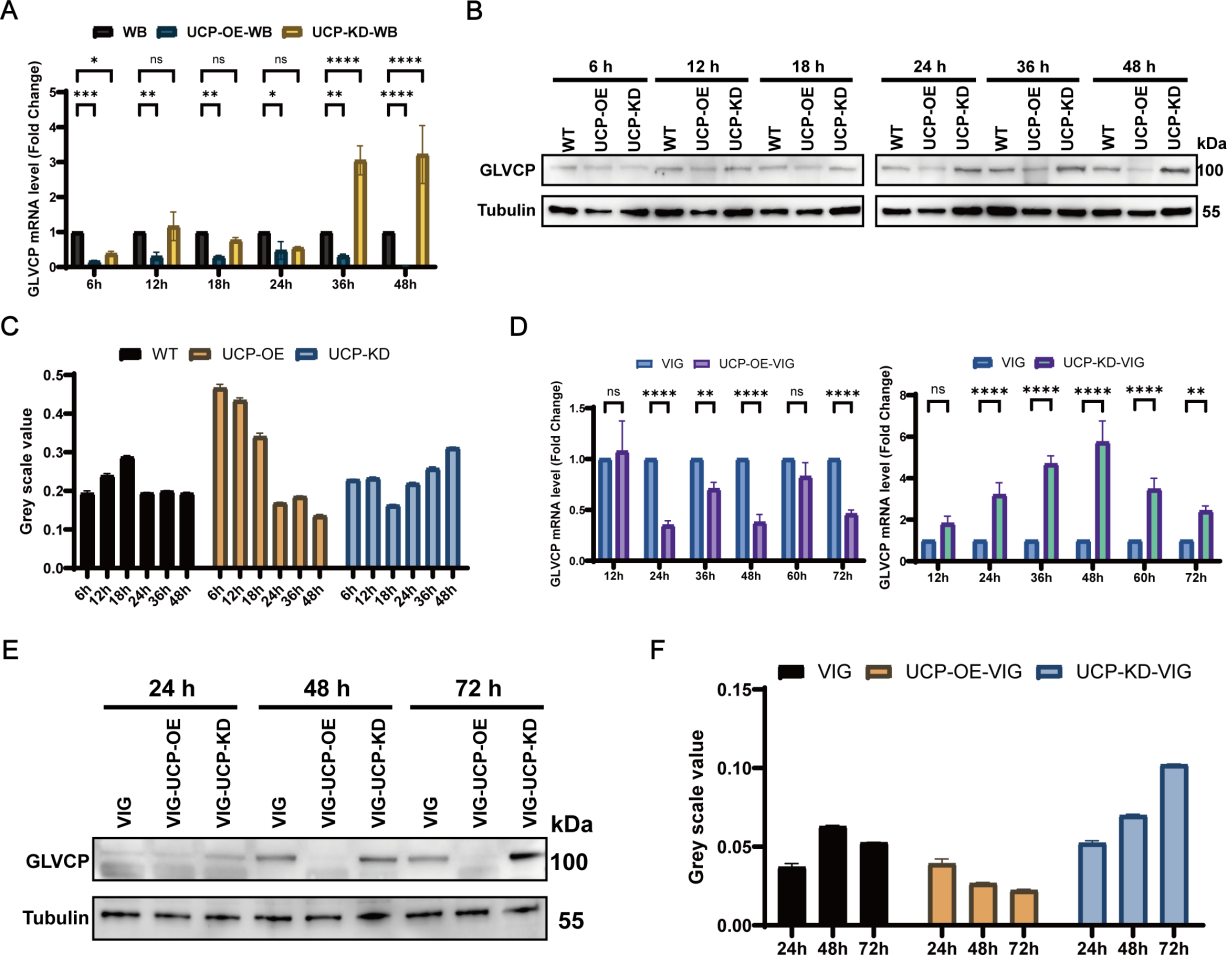
UCP regulates GLV replication in *G. duodenalis*. (**A**) The mRNA level confirmation of the relationship between GLV and UCP in the initial stage of infection. (**B, C**) Western blotting (**B**) and grayscale value analysis (**C**) confirm the relationship between GLV and UCP in the initial infection stage. (**D**) The mRNA level confirmation of the relationship between GLV and UCP in the chronic infection stage. (**E, F**) Western blotting (**E**) and grayscale value analysis (**F**) confirm the relationship between GLV and UCP in the chronic infection stage.

Following the successful development of UCP knockdown and overexpression strains, we aimed to investigate whether UCP plays a role in regulating GLV proliferation during the early stages of infection or influences viral replication in stably infected VIG strains. We infected equal numbers of UCP knockdown, overexpression, and wild-type WB strains with identical GLV inoculums and subsequently monitored GLVCP transcript and protein levels at various time points using qRT-PCR and western blot analysis. Within 24 h post-infection, both modified strains exhibited significant fluctuations in GLVCP mRNA levels, consistently exhibiting lower expression compared to the wild-type strain. This observation suggests an adaptation period for viral proliferation in *Giardia* trophozoites and indicates potential competitive or synergistic interactions with UCP modulation (Fig. 5A). Notably, UCP knockdown resulted in higher GLV mRNA levels compared to overexpression. This differential became more pronounced over time, with knockdown enhancing and overexpression suppressing viral transcription. Western blot analysis revealed that GLVCP expression was upregulated in wild-type strains peaking at 18 h before stabilizing. In knockdown strains, there was a gradual accumulation of GLV following the initial infection “shock,” whereas overexpression strains exhibited a progressive decline in viral protein expression (Fig. 5B and C). These observations collectively indicate that UCP is involved in regulating GLV replication from the early stages of infection.

To further explore UCP modulation, we examined stably GLV-infected VIG strains using both knockdown and overexpression methodologies. The qRT-PCR analysis revealed that, although GLVCP transcript levels fluctuated in UCP-knockdown strains, they remained consistently higher than those in wild-type controls. In contrast, UCP overexpression resulted in the opposite effect (Fig. 5D). Western blot analysis corroborated these findings at the protein level, showing downregulation of GLVCP expression in overexpression strains and upregulation in knockdown strains (Fig. 5E and F). Taken together, these results illustrate that UCP significantly mediates role in GLV capsid protein expression and, consequently, viral proliferation during both the initial infection phase and the established persistence phase.

### Transcriptional and metabolic differences in *Giardia* induced by GLV

**Fig 6:**
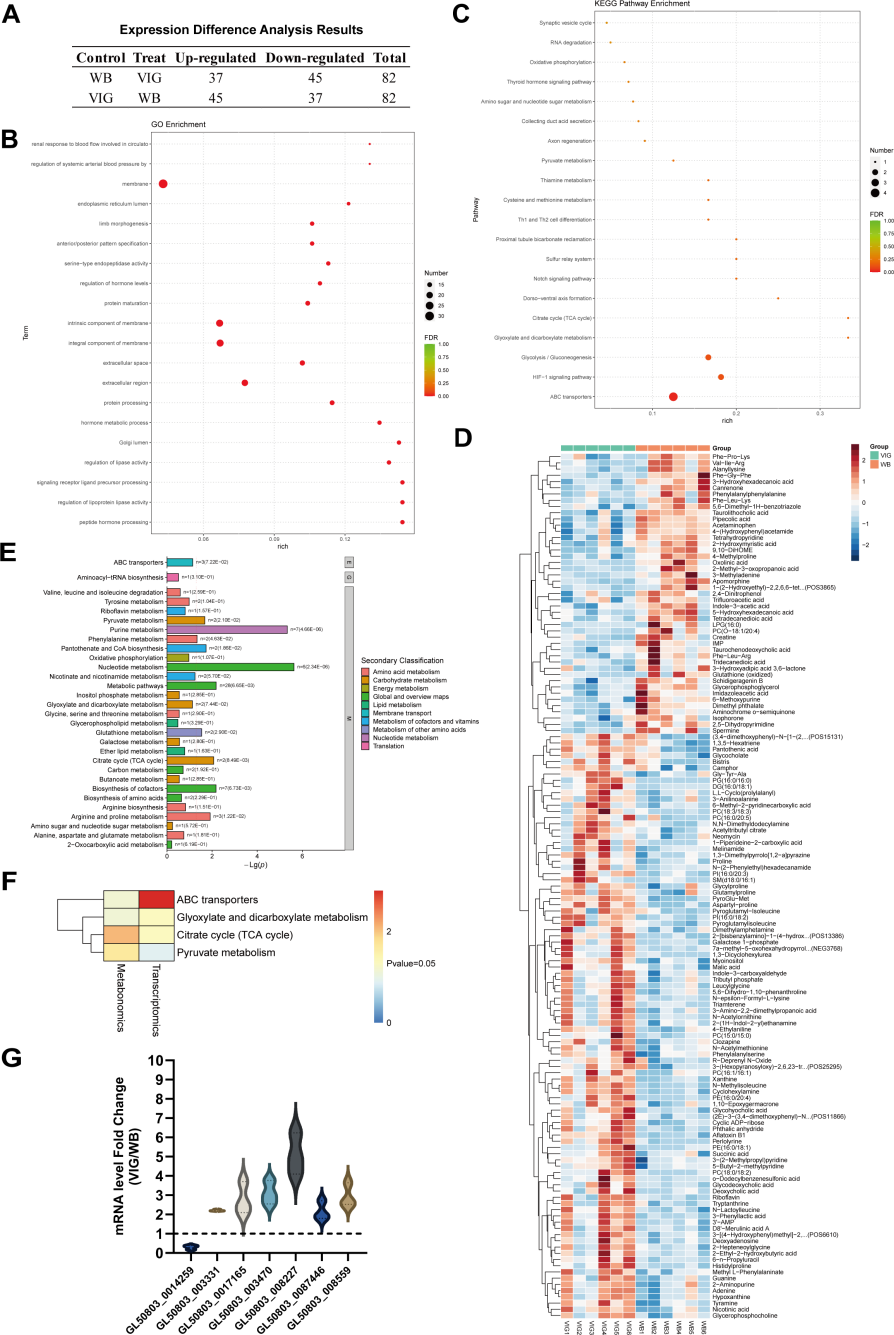
Transcriptome-metabolome correlation analysis of *Giardia* VIG and WB strains. (**A**) Results of differential gene expression between VIG and WB strains. (**B**) GO enrichment analysis of differential gene expression between VIG and WB strains. The “rich factor” refers to the ratio of the number of differentially expressed genes enriched in a specific GO term to the total number of differentially expressed genes annotated. A higher rich factor indicates a greater degree of enrichment. (**C**) KEGG enrichment analysis of differential gene expression between VIG and WB strains. The “rich factor” refers to the ratio of the number of differentially expressed genes enriched in a particular pathway to the total number of differentially expressed genes that have been annotated. A larger rich factor indicates a higher degree of enrichment. (**D**) Hierarchical clustering results of differential metabolites in the comparison group VIG vs WB. Darker shades of red indicate higher relative expression levels, while darker shades of blue signify lower relative expression levels. (**E**) Bar plot of enrichment analysis of KEGG pathways between VIG and WB strains. It highlights the significance of various biological processes and metabolic pathways, as indicated by their −log_10_ (*P*) values. (**F**) Differential metabolite statistics *P*-value heatmap from integrated transcriptomics and metabolomics analysis. The heatmap represents the magnitude of *P*-values with a color gradient, ranging from blue (indicating lower *P*-values) to red (indicating higher *P*-values). (**G**) Transcriptional level changes of shared proteins from integrated transcriptomics and metabolomics analysis. The dashed line in the graph represents the baseline for mRNA level changes (fold change = 1).

The knockdown and overexpression studies of UCP have substantiated its critical role in the replication of GLV. Considering that GLV is an obligate parasite of *G. duodenalis*, it is plausible that, in addition to UCP, other proteins within the trophozoites are implicated in the life cycle of GLV. To elucidate the relationship between trophozoite proteins and GLV, we performed comprehensive transcriptomic and metabolomic analyses on trophozoites from the VIG and WB strains. The transcriptomic analysis identified 82 proteins with differential transcription in the VIG strain, which stably harbors GLV, compared to the WB strain (Fig. 6A, Table S7). Subsequent GO and KEGG pathway analyses of the transcriptome data (Fig. 6B and C) indicated that the presence of GLV affects membrane-associated proteins, ABC transporters, the glycolysis pathway, and the HIF-1 signaling pathway in *Giardia* trophozoites. Notably, the transcriptional level of GAPDH, a pivotal enzyme in both the glycolysis and HIF-1 signaling pathways, was significantly elevated in the VIG strain relative to the WB strain. This observation suggests that GLV may modulate the glycolytic pathway in *Giardia* by up-regulating GAPDH.

In this study, we conducted an analysis of the trophozoite metabolism in the VIG and WB strains. Our findings indicated that the VIG strain exhibited 139 metabolites with significant differences compared to the WB strain (Fig. 6D, Table S8). Through KEGG pathway analysis, it was observed that the VIG strain displayed significantly enhanced metabolic activity in pathways such as purine metabolism, nucleotide metabolism, and ABC transporter (Fig. 6E). To elucidate the most prominent regulatory effects of GLV on *G. duodenalis*, we performed integrated transcriptomic and metabolomic analyses (Fig. 6F). The analyses revealed seven genes with significant differential expression within shared KEGG pathways, including ABC transporters, glyoxylate and dicarboxylate metabolism, the citrate cycle, and pyruvate metabolism (Fig. 6G). These genes comprise glucosamine 6-phosphate N-acetyltransferase (GL50803_0014259), malate dehydrogenase (MDH, GL50803_003331), v-ATP synthase subunit (GL50803_008559), and four ABC transporters (GL50803_0017165, GL50803_003470, GL50803_008227, and GL50803_0087446). These findings demonstrate GLV’s complex regulation of *Giardia* trophozoites at both transcriptional and metabolic levels, with particularly prominent effects on glycosylation and ATP metabolism.

### UCP regulates the replication of GLV by modulating glycosylation

**Fig 7:**
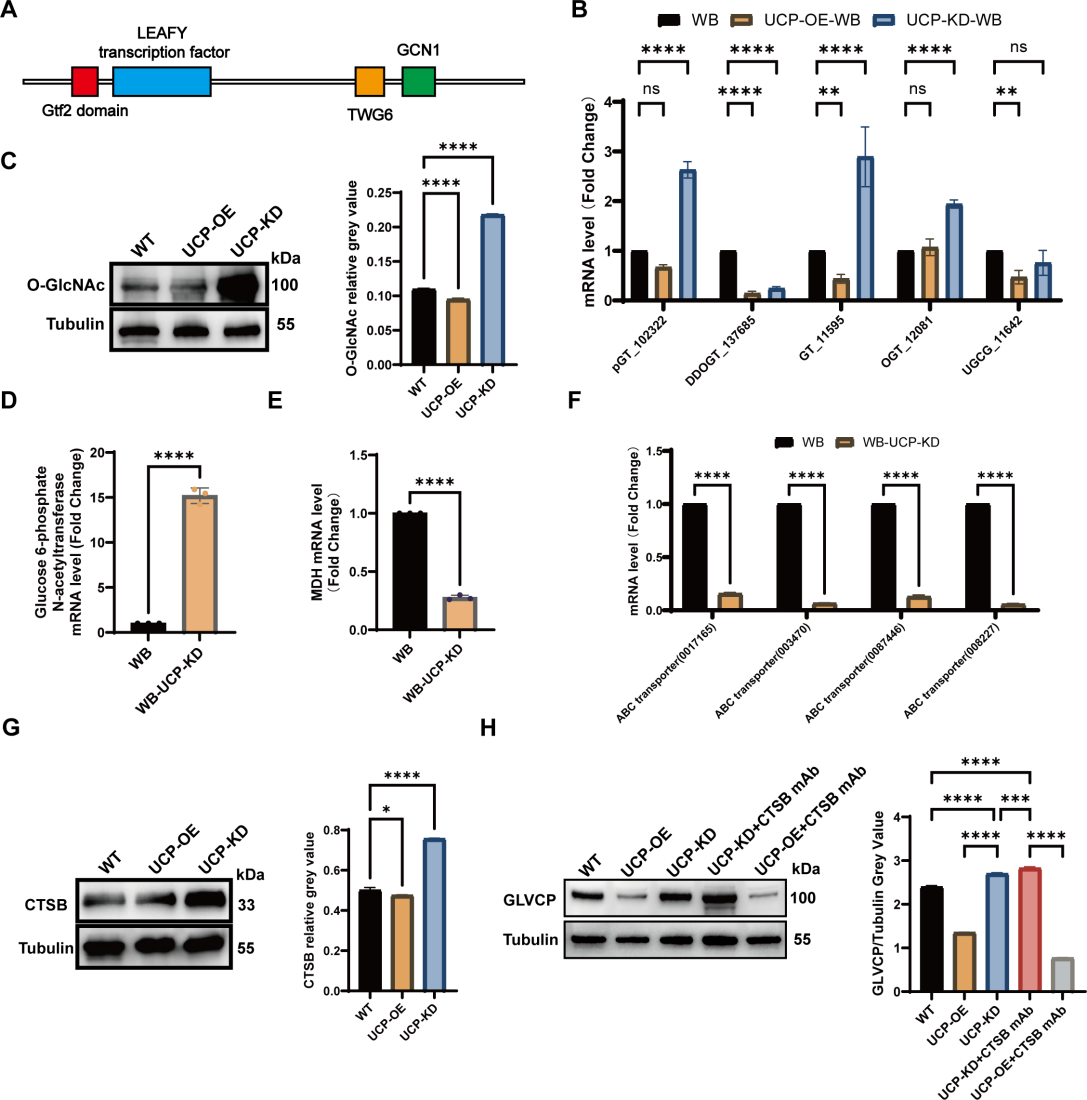
UCP regulates the replication of GLV by modulating glycosylation. (**A**) Schematic diagram of the predicted UCP functional domains by SWISS-MODEL. UCP may include four domains: Gtf2 (red), LEAFY transcription factor (blue), TWG6 (orange), and GCN1 (green). (**B**) Modulation of five *Giardia* glycosyltransferase mRNA levels by UCP knockdown and overexpression. (**C**) Effects of UCP knockdown and overexpression on O-GlcNAc expression levels and grayscale profiles. (**D, E, and F**) Regulation of Glucosamine 6-phosphate N-acetyltransferase (**D**), MDH (**E**), and four ABC transporter (**F**) mRNA levels through UCP knockdown. WB strain was used as the control. (**G**) Effects of UCP knockdown and overexpression on CTSB expression levels and grayscale profiles. (**H**) Analysis of GLVCP expression levels and grayscale values in VIG, VIG-UCP-KD, and VIG-UCP-OE strains blocked with CTSB antibody. Results are expressed as mean ± standard deviation from three separate experiments, **P < 0.01, ****P < 0.0001, with “ns” denoting “not significance” (*P* > 0.05).

Based on our previous experiments, we found that GLV induces metabolic changes in *Giardia*, while UCP affects GLV proliferation. To explore how UCP influences GLV proliferation, we employed SWISS-MODEL to analyze UCP’s amino acid sequence and predict its functional domains (Fig. 7A). Among the top 10 models, 6 consistently identified a Gtf2 domain at amino acids 23–51. Gtf2 is known to bind glycosyltransferase, stabilizing its structure and activity by preventing misfolding and aggregation. Other predicted domains included LEAFY transcription factor, TGW6, and GCN1, suggesting that UCP may function similar to Gtf2 proteins. We identified five currently published glycosyltransferase genes in *Giardia*: putative glycosyltransferase (GL50803_00102322, pGT_102322), dolichyl-diphosphooligosaccharide-protein glycosyltransferase (GL50803_00137685, DDOGT_137685), glycosyl transferase family protein (GL50803_0011595, GT_11595), O-linked GlcNAc transferase (GL50803_0012081, OGT_12081), and ceramide glucosyltransferase (GL50803_0011642, UGCG_11642). Analysis of transcriptional changes in UCP-knockdown and overexpression strains (Fig. 7B) showed that reduced transcription levels of dolichyl-diphosphooligosaccharide-protein glycosyltransferase and ceramide glucosyltransferase compared to wild-type strains. Conversely, the other three glycosyltransferases were significantly upregulated when UCP transcription was inhibited. This indicates that UCP acts as a negative regulator of glycosylation, with UCP suppression enhancing glycosylation signals in *Giardia*. Based on these findings, we examined O-GlcNAc expression, a marker of O-glycosylation modification, in *Giardia* trophozoites modified by UCP (Fig. 7C). Results showed that O-GlcNAc expression was significantly upregulated upon UCP knockdown and downregulated with UCP overexpression. This confirms that UCP regulates O-GlcNAc-mediated O-glycosylation.

To investigate whether UCP is involved in the transcriptional and metabolic changes in *Giardia* regulated by GLV, we performed qRT-PCR analyses on seven differentially expressed proteins identified through transcriptomic and metabolomic approaches in UCP-knockdown *Giardia* trophozoites. Results showed that glucosamine-6-phosphate N-acetyltransferase (GNA1) was upregulated at the transcriptional level in UCP-knockdown strains (Fig. 7D). Additionally, mRNA levels of MDH and ABC transporters were consistently down-regulated in UCP-knockdown strains (Fig. 7E and F). GNA1 is an important enzyme for UDP-GlcNAc synthesis in the pre-O-glycosylation phase. Its upregulation suggests that UCP downregulation affects both O-glycosylation and UDP-GlcNAc synthesis in *Giardia*. The downregulation of ABC transporters may be associated with organelle acidification (30) and *Giardia*’s internal homeostasis, while MDH downregulation may be related to energy metabolism inhibition by GLV to promote viral replication. Given that GLV downregulates UCP transcription (Fig. 2D), these findings indicate that GLV regulates *Giardia*’s energy metabolism by upregulating O-GlcNAc-mediated O-glycosylation via UCP, altering *Giardia*’s internal homeostasis to facilitate GLV replication.

Among the 10 potential GLV-interacting proteins identified in a previous study (Fig. 2D), Cathepsin B (CTSB_14019) was significantly upregulated in the presence of GLV, and it has been reported that CTSB can be modified by O-glycosylation. To determine whether CTSB in *Giardia* is modified by GLV-mediated O-glycosylation, thereby facilitating the cleavage of GLVCP and the release of the GLV genome, we conducted an antibody-blocking assay for analysis. The results showed that O-glycosylation mediated by UCP can regulate the protein expression of CTSB (Fig. 7G). Meanwhile, when CTSB was blocked by its antibody, the activity of cathepsin B was inhibited (Fig. 7H), leading to an upregulation in the protein amount of GLVCP, which implies weakened cleavage of GLVCP by CTSB. This indicates that CTSB is an important protein regulating GLV replication through its cleavage of GLVCP. This process affects the release of the GLV genome and viral proliferation.

## DISCUSSION

Currently, research on GLV life cycle remains limited. The RdRp and CP of GLV play important roles in the life cycle of GLV. Our laboratory’s previous studies have emphasized the interaction of DnaJ or Rab2a protein with RdRp, highlighting the key roles of those two proteins in the replication and transcription processes of GLV. Wang et al. suggested that GLV enters *Giardia* trophozoites through receptor-mediated endocytosis (24), but the viral receptor of GLV has not yet been identified. The invasion of GLV can be inhibited by regulating the pH value inside the *Giardia* trophozoites with specific reagents (31). Similarly, polyclonal antibodies against GLVCP can prevent GLV from entering *G. duodenalis*, indicating that GLVCP plays an important role in the viral invasion process (32). Notably, unlike other dsRNA viruses (33), GLV lacks prominent surface fibers for receptor binding (10). Structural analysis of GLVCP indicates that the folded GLVCP contains non-charged residues, which may facilitate viral entry (33). Given the unclear functions of GLVCP in GLV life cycle, when screening for potential proteins that interact with GLVCP, we classified these proteins based on their annotations from multiple databases (NCBI, GiardiaDB, and UniProt) and selected candidates that may affect the viral life cycle (e.g., adsorption, injection, fusion, internalization, uncoating, and release). The 48 selected candidate proteins cover a variety of functions, such as protein maturation, endosomes, membrane fusion, transcriptional regulation, and transport. This also includes some uncharacterized proteins, like the UCP that we screened out as interacting with GLVCP.

After verifying the interaction between UCP and GLVCP, we used computational predictions from AlphaFold 3 and Schrödinger software to identify key amino acid residues involved in the interaction between these two proteins, with five residues located in the N-terminus and five in the C-terminus. However, experimental results using truncation mutants revealed an unexpected increase in protein binding affinity when the C-terminal residues were deleted. This discrepancy may be attributed to several factors. The C-terminal domain may exert steric hindrance or negative regulatory effects on the interaction in its full-length form, thereby inhibiting binding (34, 35). Upon truncation, the removal of this domain could lead to conformational changes that expose additional binding sites or enhance binding affinity (36, 37). Additionally, experimental conditions, such as buffer composition, protein concentration, temperature, and pH, may influence protein conformation and binding capacity, contributing to the divergence between computational predictions and experimental observations (38). The inherent limitations of computational models, which may not fully capture the dynamic and complex interactions of proteins in a cellular environment, should also be considered. Future studies could include progressive truncation of the C-terminal domain to identify critical residues involved in binding. Moreover, examining post-translational modifications on the C-terminal domain and conducting experiments under conditions that more closely mimic the cellular environment could provide deeper insights. These approaches will help us understand the mechanisms underlying the effects of C-terminal truncation on protein binding and improve the accuracy of protein interaction predictions.

When we conducted domain analysis on UCP, the domains that we identified as potentially present, such as LEAFY transcription factor, TGW6 (39), and GCN1 (40, 41), were not relevant to *Giardia*. The small Gtf2 protein region (42) that UCP contains, however, may be a key functional domain for a Gtf2 protein. The changes detected in the five glycosyltransferases, as well as the upregulation of O-GlcNAc protein expression levels, also validated our hypothesis. Meanwhile, the upregulation of transcription levels of other glycosyltransferases may be a compensatory effect of enzymes with similar functions within the same family to maintain glycan synthesis (43). Among the seven significantly different proteins identified by the combined analysis of transcriptomics and metabolomics, GNA1 (44, 45), which is an important enzyme upstream in the O-glycosylation process (46, 47), was upregulated in the case of UCP knockdown. This indirectly indicates that UCP, as a candidate protein regulating glycosyltransferases, has a comprehensive regulatory effect on the glycosylation of *Giardia* (especially O-glycosylation). This also indicates that the glycosylation regulation of *Giardia* by GLVCP is multifaceted, including the regulation of ABC transporters to mediate endosome acidification, thereby influencing viral proliferation and replication (30, 48–50). It is worth noting that, as an important participant in the tricarboxylic acid cycle, MDH regulated by GLV-mediated glycosylation may cause *Giardia* to shift its intracellular environment towards inhibiting aerobic metabolism (tricarboxylic acid cycle) (51) and promoting anaerobic regulation (HIF-1 signaling pathway) (52), providing a more suitable environment for GLV survival.

In addition to these proteins, while screening for potential GLVCP-interacting proteins, we identified proteins that showed significant differences under the influence of GLV, such as CTSB. Reovirus infects mammalian cells via endocytosis and entry into the endoplasmic reticulum (53, 54). The outer layer of the virus particles is processed by CTSB, generating infectious subviral particles (ISVPs). These ISVPs complete the infection through further conformational changes and membrane penetration mechanisms (55). Importantly, CTSB can undergo glycosylation modifications, particularly O-GlcNAc-mediated glycosylation. Our Western blot results confirmed that UCP-mediated O-glycosylation promotes CTSB expression (56). Antibodies against CTSB blocked its proteolytic activity (57), significantly increasing GLVCP levels. CTSB processes the viral outer layer or capsid proteins by enzymatic cleavage, facilitating viral genomic RNA release for replication and transcription, which is similar to *Reovirus* (58). Our results elucidate part of the GLV life cycle: GLVCP interacts directly with UCP to downregulate its transcription and expression. This promotes O-glycosylation in *Giardia* trophozoites, regulates ATP transport, and mediates CTSB activity (59), facilitating GLVCP cleavage (60) and viral genome release (61). However, it remains unclear whether the cleavage of GLV by cathepsin B occurs during the GLV invasion or during the degradation of GLV proteins by *Giardia*. Additionally, it is yet to be determined whether the GLVCP cleavage is part of a dynamic equilibrium initiated by GLV itself or a result of *Giardia*’s defense mechanisms against GLV infection. These questions remain to be addressed.

## Data Availability

No new data were created or analyzed in this study. All data associated with this study are provided in the supplemental material.

## SUPPLEMENTAL MATERIAL

Supplemental figures jvi.00883-25-s0001.docx Figures S1 to S5.

Supplemental text jvi.00883-25-s0002.docx Supplemental methods.

Supplemental tables jvi.00883-25-s0003.pdf Tables S1 to S4 and S6.

Table S5 jvi.00883-25-s0004.xlsx Screening of *Giardia duodenalis* membrane and cytoplasm proteins.

Table S7 jvi.00883-25-s0005.xlsx Significantly differentially expressed proteins in the transcriptomic comparison between the VIG strain and the WB strain.

Table S8 jvi.00883-25-s0006.xlsx Significantly differentially expressed metabolites in the metabolomics comparison between the VIG strain and the WB strain.

ASM does not own the copyrights to Supplemental Material that may be linked to, or accessed through, an article. The authors have granted ASM a non-exclusive, world-wide license to publish the Supplemental Material files. Please contact the corresponding author directly for reuse.
