# Supplementary material for: Identification of an uncharacterized protein as a novel regulator of Giardia lamblia virus (GLV) infection in Giardia duodenalis
Source: J Virol. 2025 Sep 18;99(10):e00883-25. doi: 10.1128/jvi.00883-25 (PMC12548405; doi:10.1128/jvi.00883-25)
Supplement: Supplemental figures — Figures S1 to S5. [file jvi.00883-25-s0001.docx]

**Supplemental Figure Legend**


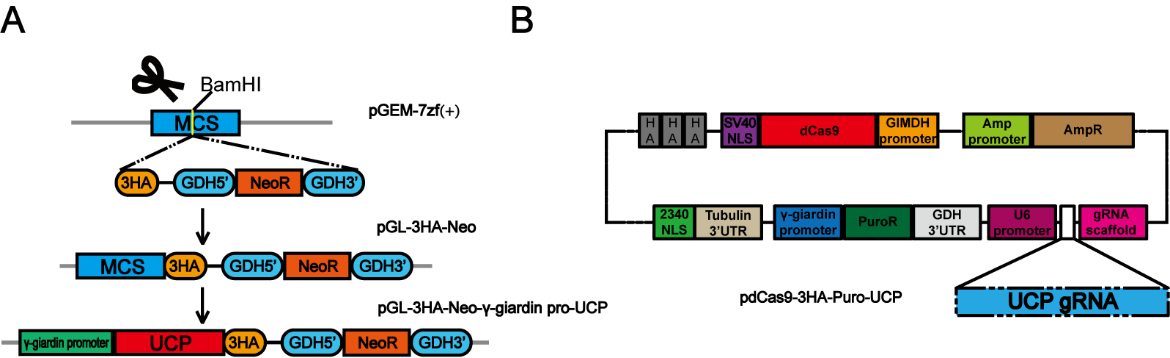


**Fig S1. Schematic diagrams of the construction of overexpression and knockdown plasmids.** **(A)** Construction of overexpression plasmid. The promoter, HA tag, and resistance gene were inserted into the pGEM-7zf (+) plasmid via Gibson assembly. **(B)** Construction of knockdown plasmid. The gRNA of UCP was inserted into the cassette of the dCas9g1pac plasmid through restriction enzyme digestion and annealing.


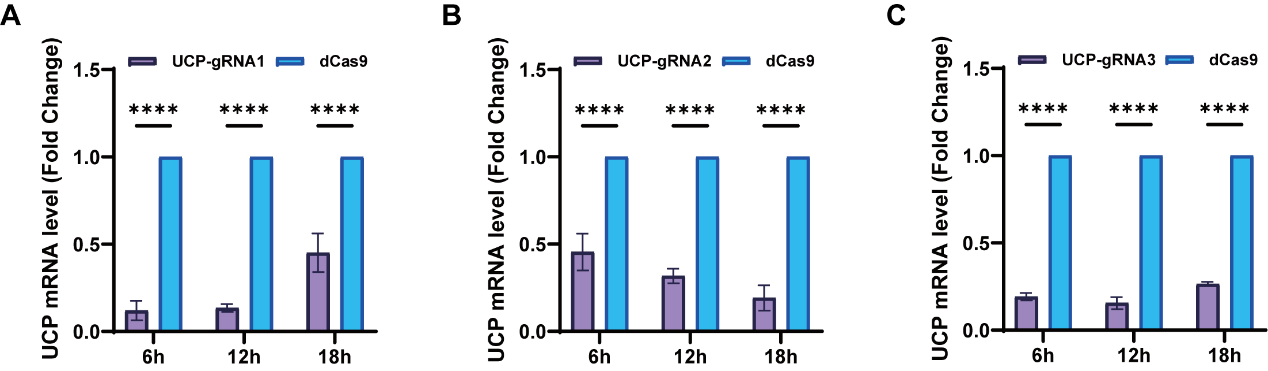


**Fig S2. Screening for optimal gRNAs targeting UCP. (A-C)** WB strain trophozoites were electroporated with UCP-targeting gRNA knockdown plasmids (gRNA1-3) at a ratio of 20 μg plasmid per 106 trophozoites. UCP expression levels were then measured at 6 h, 12 h, and 18 h post-electroporation, with dCas9g1pac-electroporated trophozoites serving as controls. Results are expressed as mean ± standard deviation from three separate experiments, ****p<0.0001.


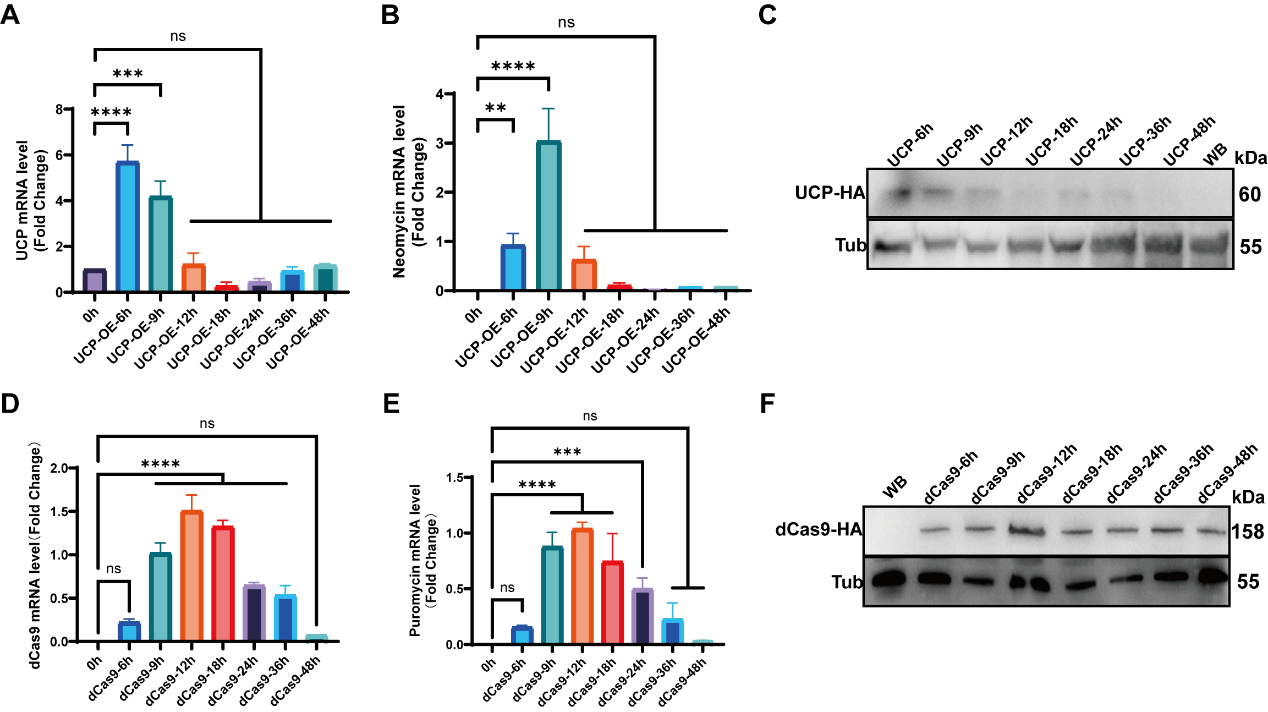


**Fig S3. Establishment of appropriate time points for drug screening.** **(A-C)** After electroporation of UCP-overexpression plasmid (20 μg/106 WB trophozoites), parallel qRT-PCR analysis of UCP (A) and G418/neomycin (B) transcripts and Western blot detection of exogenous UCP protein (C) were conducted at 6-48 h, with native WB trophozoites serving as controls. **(D-F)** WB strain trophozoites (1×106 cells) were electroporated with 20 μg of the dCas9g1pac plasmid. The dCas9 (D) and puromycin transcript levels (E) and dCas9 expression (F) were analyzed by qRT-PCR and western blot at 6 h, 9 h, 12 h, 18 h, 24 h, 36 h, and 48 h post-transfection, using native WB trophozoites as controls. Results are expressed as mean ± standard deviation from three separate experiments, **p<0.01, ***p<0.001, ****p<0.0001, with "ns" denoting "not significance" (p>0.05).


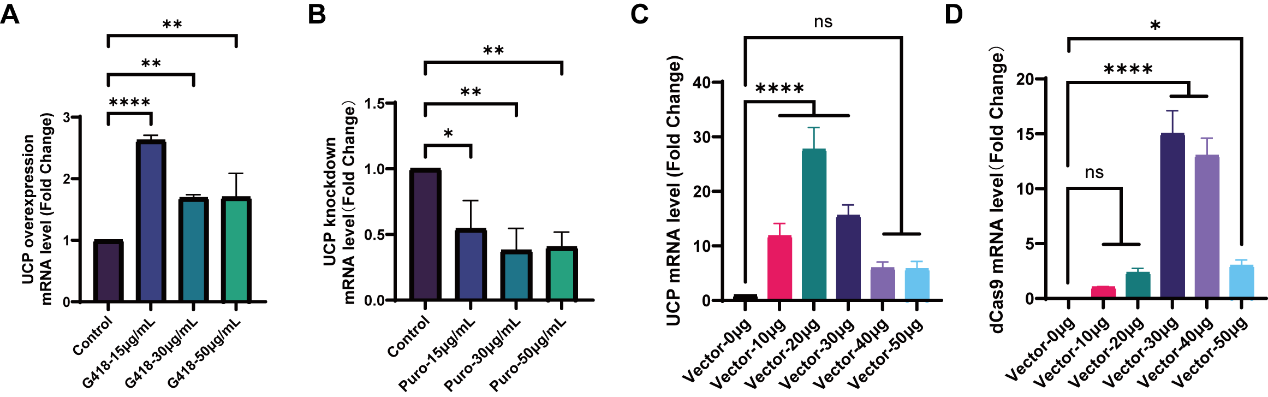


**Fig S4. Optimization of drug concentration screening and plasmid transfection dosage**. **(A, B)** UCP mRNA levels in WB trophozoites post-electroporation (20 μg plasmid/106 cells) under escalating drug selection (15-50 μg/mL) (puromycin for knockdown strains, G418 for overexpression strains). **(C, D)** UCP transcription levels in drug-selected WB trophozoites after electroporation with gradient plasmid concentrations (10-50 μg/106 cells). Antibiotic selection: 30 μg/mL puromycin (knockdown) or 15 μg/mL G418 (overexpression). Results are expressed as mean ± standard deviation from three separate experiments, **p<0.01, ***p<0.001, ****p<0.0001, with "ns" denoting "not significance" (p>0.05).


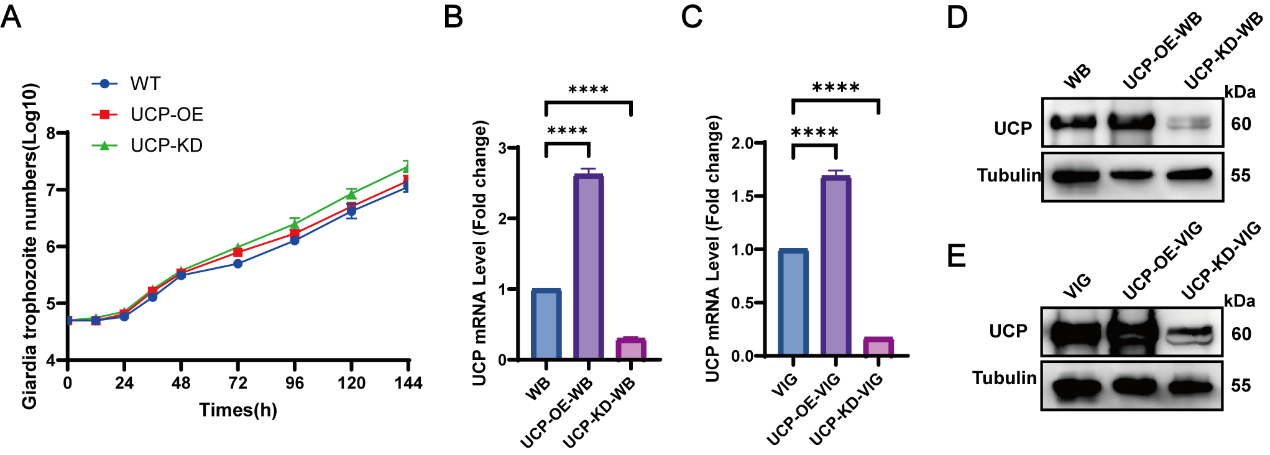


**Fig S5. The proliferation efficiency and editing efficiency of the UCP-modified strain.** **(A)** Growth curves of G. duodenalis strains (WB, UCP overexpression, and UCP knockdown) were monitored over a 7-day period. The UCP overexpression and knockdown strains exhibited distinct proliferation patterns compared to the wild-type WB strain. OE means overexpression. KD means knockdown. Data are represented as mean ± standard deviation (n=3 independent experiments). **(B, C)** Transcriptional levels of UCP in UCP knockdown and overexpression strains were examined in both WB and VIG strains. **(D, E)** UCP expression in UCP knockdown and overexpression strains were examined in both WB and VIG strains. Results are expressed as mean ± standard deviation from three separate experiments, ****p<0.0001.
