# Supplementary material for: Identification of an uncharacterized protein as a novel regulator of Giardia lamblia virus (GLV) infection in Giardia duodenalis
Source: J Virol. 2025 Sep 18;99(10):e00883-25. doi: 10.1128/jvi.00883-25 (PMC12548405; doi:10.1128/jvi.00883-25)
Supplement: Supplemental text — Supplemental methods. [file jvi.00883-25-s0002.docx]

#### Supplemental material

#### Production of polyclonal antibodies

For rabbit anti-GLVCP, rabbit anti-UCP, and rabbit anti-Cathepsin B (anti-CTSB) polyclonal antibody production, 8-week-old New Zealand rabbits were immunized subcutaneously on days 0, 14, and 21 with 500 μg of purified recombinant GLVCP and UCP proteins. Before immunization, the purified proteins were emulsified with Sigma Freund's complete adjuvant for the primary immunization (Day 0) (Sigma-Aldrich, Cat. #F5881) and with Freund's Incomplete Adjuvant for the booster immunization (Day 14) and final immunization (Day 21) (Sigma-Aldrich, Cat. #F5506). A week later, the rabbits were euthanized and total blood was withdrawn by cardiac puncture. The resulting polyclonal serum were then obtained and stored at -20℃.

#### Identification of membrane and cytosol proteins of *Giardia* trophozoites

According to the instructions of the Membrane and Cytosol Protein Extraction Kit (YEASEN, China), the membrane and cytosolic proteins of approximately 10^7^ *Giardia* trophozoites of WB strain were separated. The separated products were analyzed by SDS-PAGE. Meanwhile, the products were sent to SHANGHAI BIOPROFILE TECHNOLOGY Co., Ltd. for large-scale protein identification (Shotgun).

#### Identification of proteins immunoprecipitated with GLVCP by MS/MS

The protein samples immunoprecipitated with GLVCP were sent to Sangon Biotech (Shanghai) Co., Ltd. for analysis. The database used for the search was the UniProt reference proteome database for *Giardia lamblia*.

#### Negative-staining transmission electron microscopy (Negative-staining TEM)

For negative staining of virions, 10 µL of purified viral pellet was immediately loaded onto carbon-coated 400-mesh copper grids and allowed to adsorb for 2 min at room temperature. The grids were then negatively stained with 20 µL of 3 % (w/v) phosphotungstic acid (pH 7.0) for 5 min, blotted to remove excess stain, and air-dried. Imaging was performed on a Hitachi HT-7800 transmission electron microscope operated at 120 kV.

#### Scanning electron microscopy (SEM)

*Giardia* trophozoites were fixed overnight at 4 °C in 2.5 % (v/v) glutaraldehyde. After gentle transfer to poly-L-lysine-coated glass slides to secure the specimens, they were dehydrated through an ascending ethanol series (30 %, 50 %, 70 %, 90 %, and twice in 100 %), followed by two exchanges of 100 % amyl acetate for complete solvent substitution. Critical-point drying was then performed with supercritical CO₂ (Leica EM CPD300). The dried samples were mounted on aluminum stubs with carbon-conductive adhesive tabs and sputter-coated for 30 s with a 5–10 nm Au–Pd (80 : 20) layer (Quorum SC7620). Imaging was carried out using either a HITACHI Regulus 8100 cold-field-emission SEM or a HITACHI SU8100 SEM operated at 5 kV, 8 mm working distance, and 10 µA emission current.

#### *Giardia* proliferation assay

To clarify the proliferation of the constructed VIG strain, we inoculated VIG strain, VG strain and WB strain into culture tubes at a density of 5×10^4^ trophozoites/tube. Trophozoites were cultured at 37℃ for 12 h, 24 h, 36 h, 48 h, 72 h, 96 h, 120 h, and 144 h, and then collected and counted.

Similarly, to clarify the proliferation of the constructed WB strain with overexpressed UCP plasmid and the WB strain with UCP knockdown plasmid, we inoculated WB strain, the WB strain with overexpressed UCP, and the WB strain with UCP knockdown into culture tubes at a density of 5×10^4^ trophozoites/tube. The trophozoites were cultured for 24 h, 48 h, 72 h, 96 h, 120 h, and 144 h, and then collected and counted. For each strain, three replicates were performed at each time.

#### Evaluation of gRNA-mediated knockdown efficiency

To confirm the knockdown efficiency of the gRNAs, we subcloned the three designed UCP-specific gRNAs into the dCas9g1pac vector. Three UCP knockdown plasmids (gRNA1-3) were then electroporated into the same number (1×10^6^ trophozoites) of *Giardia* trophozoites. Total RNA was collected from the trophozoites at 6 h, 12 h, and 18 h post-electroporation. The knockdown efficiency of the UCP knockdown plasmids was determined by qRT-PCR, which will be used for subsequent experiments.

#### Optimization and validation of time points for drug screening

To determine the appropriate time points for drug screening in overexpressing and knockdown strains, we transiently electroporated overexpressing and knockdown plasmids into the same number (1×10^6^ trophozoites) of trophozoites, respectively. Trophozoite proteins and total RNA were collected at 6 h, 9 h, 12 h, 18 h, 24 h, 36 h, and 48 h post-electroporation. The changes in transcriptional and protein expression levels of the transiently transfected overexpressing and knockdown strains were then determined by qRT-PCR and Western blot.

#### Optimization of drug concentration screening

After confirming the optimal drug screening timepoints for both knockdown and overexpression strains, we proceeded to determine appropriate drug concentrations targeting UCP-modified *Giardia* strains. For knockdown strains, puromycin (15 μg/mL) was added 12 h post-electroporation of the knockdown plasmid, while G418 (15 μg/mL) was administered 6 h after electroporation of the overexpression plasmid. Cultures were maintained statically at 37°C, with trophozoite proliferation monitored at 12 h intervals. When trophozoite density reached 50% confluency, the medium was replaced with fresh solution containing incrementally higher antibiotic concentrations (puromycin or G418). At each concentration point, we collected more than 1×10^7^ trophozoites for total RNA extraction, followed by UCP transcript level quantification using qRT-PCR. All drug concentration tests were performed in triplicate to ensure data reliability.

#### Determination of optimal plasmid transfection dosage

After establishing the optimal drug screening time and concentration parameters, we investigated the effect of plasmid dosage on electroporation efficiency by transfecting *Giardia* trophozoites with varying amounts of knockdown and overexpression plasmids. Transfected cells were cultured under selection with puromycin (30 μg/mL) or G418 (15 μg/mL) in a static 37°C incubator, with trophozoite proliferation monitored at 12-hour intervals. For each plasmid dosage tested, we collected more than 1×10^7^ trophozoites for total RNA extraction, followed by qRT-PCR analysis to determine UCP and dCas9 transcript levels. All plasmid dosage experiments were performed in triplicate to ensure data reproducibility.

#### Antibody blocking assay

Viable VIG *Giardia* trophozoites were collected from culture and prepared into three groups, each containing 1 × 10^6^ trophozoites. Additionally, *Giardia* trophozoites with UCP-knockdown (VIG-UCP-KD) and UCP-overexpression (VIG-UCP-OE) were collected and prepared into six groups each, comprising 1 × 10^6^ trophozoites per group. Subsequently, three groups of both VIG-UCP-KD and VIG-UCP-OE trophozoites were each treated with 2 mL of anti-CTSB serum. All samples were then incubated at 37°C for 6 h. After incubation, protein samples were collected from each group of trophozoites and analyzed by Western blot to investigate the differences in specific protein expression among *Giardia* trophozoites with different genetic modifications.
