# Supplementary material for: Identification of an uncharacterized protein as a novel regulator of Giardia lamblia virus (GLV) infection in Giardia duodenalis
Source: J Virol. 2025 Sep 18;99(10):e00883-25. doi: 10.1128/jvi.00883-25 (PMC12548405; doi:10.1128/jvi.00883-25)
Supplement: Supplemental tables — Tables S1 to S4 and S6. [file jvi.00883-25-s0003.pdf]

Table S1. Primers used for PCR

Primer name	Primer sequence (5’-3’)	Purpose
3HA-F	GTTCGAAATCGATAAGCTTTATCCTTATGACGTCCC	For pGL-3HA-Neo vector construction
3HA-R	GATCCTTAAGCGTAATCTGGTACGTCGTATG	
Gneo-F	GACGTACCAGATTACGCTTAAGGATCCAAGCTTCACGTCGTCGTTC	
Gneo-R	CGCGTTGGGAGCTCTCCCAGCTGATCGGC	
pGL-NEO-giardin-pro-F	GACGTCGCATGCTCCTCTAGAGTGATCAAATGCCTTCGAGC	For pGL-3HA-Neo-giardin promoter vector construction
pGL-NEO-giardin-pro-R	GGGTACCGAATTCCTCGAGTTTCTTGTTTTAGGGTTAGT	
3HA-GLVCP-F	TGGTACCGAGCTCGGATCCGCCACCATGGTGTGGGGGACTGGATATGGGA	Co-IP
3HA-GLVCP-R	TTAAACGGGCCCTCTAGACTCGAGTCACAGCTTGGCTCGTGTCGTCATAAGG	
3.1UCP-NFLAG-F	GCTTATGTCCAGTGTGGTGGAATTCATGACAGCCGTTGAATACAAG	
3.1UCP-NFLAG-R	TTAAACGGGCCCTCTAGACTCGAGTTACTCGAGACCCGCAGACGCTAC	
3.1-UBISEG1-F	CTTATGTCCAGTGTGGTGGAATTCCTCTACTACAAGAATAAGAAC	
3.1-UBISEG2-R	AGAGTTGCGTGTGACAAGCTTGAAGTTCTTATTCTTG	
3.1-UBISEG2-F	AACTTCAAGCTTGTCACACGCAACTCTTACCTCTTTGTCCTCAAG	
3.1-UBISEG3-R	GAAGACCTTGCGGAGGTAAGAGTTGCGTGTTACGAA	
3.1-UBISEG3-F	TCTTACCTCCGCAAGGTCTTCAACGCGAAGTCCAATATGCA	
3.1-UBISEG4-R	TCGTCCTGCGTCGTCCCCTCATTAGGGAGCTCGATCTTTCTCAT	
3.1-UBISEG4-F	CGAGCTCCCTAATGAGGGGACGACGCAGGACGAGCAGCTAAAG	
3.1-UBISEG5-R	CGGGCCCTCTAGACTCGAGTTAAACATCCATCTCAACCTTGCGAAGG	
Bivc155-UCP-F	GCCATGGAGGCCCGAATTCGGATGACAGCCGTTGAATACAAGGAG	BiFC
Bivc155-UCP-R	GATTTTGCACGCCGGACGGGTACCCTCGAGACCCGCAGACGCTACAA	
Bivn173-GLVCP-F	GCCATGGAGGCCCGAATTCGGGCCACCATGGTGTGGGGGACTGGA	
Bivn173-GLVCP-R	TTGCACGCCGGACGGGTACCCAGCTTGGCTCGTGTCGTCATAAGG	
Bivc155-UCP-T3-F	GGCCATGGAGGCCCGAATTCGGATGACAGCCGTTGAATACAAGGAG	
Bivc155-UCP-T3-R	TTTGCACGCCGGACGGGTACCCTCGAGACCCGCAGACGCTACAACG	
Bivc155-UCP-T4-F	TGGCCATGGAGGCCCGAATTCGGATGACAGCCGTTGAATACAAGGAGA	
Bivc155-UCP-T4-R	GATTTTGCACGCCGGACGGGTACCAACATCCATCTCAACCTTGCGAAG	
Bivc155-UCP-T5-F	TATGGCCATGGAGGCCCGAATTCGGATGACAGCCGTTGAATACAAGGAG	
Bivc155-UCP-T5-R	TTTTGCACGCCGGACGGGTACCCTCGAGACCCGCAGACGCTACAACGCCG	
28a-UCP-F	TGGTGGTGGTGGTGCTCGAGCTCGAGACCCGCAGACGCTACAACG	Antibody Preparation
28a-UCP-R	GGACAGCAAATGGGTCGCGGATCCATGACAGCCGTTGAATACAAGG	

28a-GLVCP-F	CAGCAAATGGGTCGCGGATCCATGGTGTGGGGGACTGGATAT	
28a-GLVCP-R	GGTGGTGGTGGTGCTCGAGTAATCTCCTGTTACTGAAGC	
pGL-Neo-giardin pro-UCP-F	ACCCTAAAACAAGAAACTCGAGATGACAGCCGTTGAATACAAG	UCP overexpression
pGL-Neo-giardin pro-UCP-R	CATAAGGATAAAGCTTATCGATCTCGAGACCCGCAGACGCTAC	
UCPdcas9-gRNA1F	CAAAGAACGAGGATGTCTCTTCGA	
UCPdcas9-gRNA1R	AAACTCGAAGAGACATCCTCGTTC	
UCPdcas9-gRNA2F	CAAAGTGGAGTCTGTCTTCTACTA	
UCPdcas9-gRNA2R	AAACTAGTAGAAGACAGACTCCAC	UCP knockdown
UCPdcas9-gRNA3F	CAAAGAGCTACACTTACGAATTCA	
UCPdcas9-gRNA3R	AAACTGAATTCGTAAGTGTAGCTC	

Table S2. The sequence of the UCP gRNA

gRNA name	Sequence (bp)	Target site (bp)
UCP-gRNA1	GAACGAGGATGTCTCTTCGATGG	942-961
UCP-gRNA2	GTGGAGTCTGTCTTCTACTACGG	313-332
UCP-gRNA3	GAGCTACACTTACGAATTCAAGG	129-148

Notes: The underlined part is the PAM sequence.

Table S3. Primers used for qRT-PCR

Gene ID or name	Forward primer (5'-3')	Reverse primer (5'-3')
GL50803_006430	CTGCCCTCAACGCCTACAACTC	AGTTCAGAGCCAGCCCGAGAC
GL50803_0021423	TCCGCTGACCGCCGAGAC	AGATTGGTAGCAGGTTGGACTTGTC
GL50803_00103709	GTGAAGACAGCGTACAAGGAGATGG	CGGAGCGGAACGAGGAGAGG
GL50803_0014019	TTGACATACCCGCCATGATGAAGG	GTCGTCGTCTGTGCCGTAGC
GL50803_0017195	GGCAACCTCAGCATCATCTCCAAG	AGAGTCCGACGCCTTCTCAACC
GL50803_0014373	CGCCGTCATTCTTGCTGTCTCTG	CACCACCGTTGCCTCTGTTCC
GL50803_003256	AACAACAACAACAGCAGCCACAAG	GCGGGCATCGTTGAAGGGAAG

Gene ID or name	Forward primer (5’-3’)	Reverse primer (5’-3’)
GL50803_0017090	ACCGAGGATGATCTGAAGCAAATGG	GGGCGAGCGACATGATGAGTTC
GL50803_007188	GGAACTGCTGGCTTGACCTGTG	TGTCGCTCAACGCACTGGTTATC
GL50803_007569	CCAACAGCCGACATCACAGGAG	GCGATGGATCTGCGGAGTCAAC
GL50803_007532	CTACCATCCTGTTCGTTCGCCTAAG	GCTGCGGTGGCTCCTATTGC
GL50803_00101326	GGTCATGGAGCAAGTCGTGGAAC	ATTGGTTGGGTATGCTGCGTAAGG
GL50803_0016869	ACTTGCCGTGCTTCCTTGAACAG	GCCGAACTTCCCATTAGACGATCC
GL50803_005795	TCTACGACATCCTCAAGTGCCTCTC	GCCCTGCCGAACTCACCATTG
GL50803_0010892	CGCAAGGAAGACAAGGATGACTACC	CGTCTCGTCTATGTCGTTAGCCATG
GL50803_008917	ACCGCCGCCAGGGAATTATTTAC	GCCAGCACCGACAACACGATC
GL50803_007537	ATGATGCCGACGACAAGGATGAC	ATGCTGCCACGACGACTGC
GL50803_008619	ACGAGCAATCAGATGATGAGGTTCC	GACGGACAACAGCACAGGTAGC
GL50803_0016353	GGAGGACATGGACACCGAGGAC	GTGGCGTGGTTGAGCGATAGC
GL50803_0017255	GCCGTTGTCCACCACCCTTG	GTTATCCTCTCCTGACTCGCATCTG
GL50803_0032658	AGGTTGACGGAGACAGGAAGGAG	GCTCTACGGCATTAGTGACACCAG
GL50803_0061270	TGGTCAAGCAGCGTGTCAAGAG	AGAGCACCATGAGAGCAAGTAATCC
GL50803_001695	GCGAGGTCACGACGGAGGAG	CGACGACGGCTAGTTCAGTAACG
GL50803_009558	TGAAGACATGGCTGAAGGAGATTGC	AGCATTGATGGAACCCGCACTATC
GL50803_0016636	CCAGCGGCGGACACCATTATATC	GGGCACCTGAAGGGTCTTGTTG
GL50803_007244	CGCCAGACGACCTTGCTAGATG	CGACTGCGTAGAGGTGCTCATAG
GL50803_5744	GCCATCCGTTCAGCAACAATATCC	ACCTCCGCCCAAGTACCTAGC
GL50803_0091919	AGCGAAGGTGACTGGTGGAGAG	TCGGCAAACCCAAGGCAAGAAG
GL50803_0011992	TATCACCGCACAATGGCAGAAGTC	GTGACTGACGCTCTTACGCATCC
GL50803_0016124	GCATGGTGTGGCGTTTAAGAAGAC	CCGTTAGTCGTATCTCTGCGTTGTC
GL50803_006812	GCAGTTGTTCCGCCTCGCTAC	CGTTTCGCTGACCAAATTGTCTGTG
GL50803_0016934	CGGTGTTCGGCTCATCTCAACTG	AGACGCTATCTCCTCTTGGCTCATC
GL50803_0087577	TCCTTCATCCTCCCTACAGCATTGG	TGTCCGTTCCTATCAGTCTCTCGTC
GL50803_009827	GCTCGCCGCCTGGATTGC	CGCCTGAGAACCATATAGACCTTCG
GL50803_0013561	AGCGAGTCAGACGACGAGGAG	GCGTTCTTCGGCTTCTTCTTGTTC
GL50803_006242	ATCACCTCCACCGACGACCTC	AACTCTGCCTCCGCAAGATTGTAG
GL50803_0015427	TCAACGAGATCAGGGCGACTAAGG	CGTGGGAGGGCTGTCAGGAAC
GL50803_0016507	GGGCTAACATGACCATTGGAGACAG	CAAAGGCAAATCGCTGGCTGAATC
GL50803_0023300	GACAGATGACAATGCCGTGAAAGC	CTCACAGCAATAGAAGCGTTGAAGC
GL50803_007110	CAAGGCGAAGATCCAGGACAAGG	GGAGGCGGAGGACGAGGTG
GL50803_0094463	TCGTCTCCTCGCTCACTTCTATCC	CGTCGTCCCAACATCCATCTCAAC
GL50803_0012216	TCCATCGTCGGTTCTGAAGGTCTC	TCGTCTGGTGTTGAAGTCCTGTTTC
GL50803_007323	CGACTGTTCCGAAGGCATGAGAG	GACTTGTGACCTGGACGATGAAGAG
GL50803_00100864	GGAGGTGCCCGCTACGAGAC	CTTCCATACTGAGGTTGCTGCTGAG
GL50803_003582	AGGGAGGCAGCGACCAAAGAG	GGTTGCGTATTGCTCGGAGTCC
GL50803_0010429	TGAAGAACATGGGCGGTGGAATG	TTGTCGTCGTCCTCCTCATCTCC
GL50803_0010822	TGCTCTTTCGTTCGTCCATCCTTG	ACCTCTCCGATTGTCCTTCCACTC
GL50803_0033762	GGGTTTACAGTGCTCGGCTCAG	GTTGTCGTTGGTGATGGCTTGAAC
18S rRNA	GCGATCAGACACCACCGTATTCC	CCGTCAATGCCTTCAAGTTTCAGC
*Gl*Actin	CAGAACTGGCGTCAAACGTG	TTTCCTCCATACCACACGGC

Gene ID or name	Forward primer (5'-3')	Reverse primer (5'-3')
GLVCP	GCTTTTGCCCTCGTCTACC	ATCCCACACGCTCTTGACTT
Puromycin	CACCGAGCTGCAAGAACTCTTCC	CTCTCCGGCGTGGTCCAGAC
Neomycin	CGACCACCAAGCGAAACATC	TGATATTCGGCAAGCAGGCA
dCas9	AGAAGATGGATGGGACGGAAGAG	AGTGGATTTGATGTGGAATGCTACC
GL50803_00102322	AAGTTCTCACCAATACGCTTCAGG	TTCGCAGCACTCCATCACAAC
GL50803_00137685	GCACCTATTCACGAGCACAACTAC	GATACGACAGCTTGAGCAACAGAC
GL50803_0011595	GCCTGATGTGAAGTGGTGGTATG	GTCTGCGGGAGTGGGATGAG
GL50803_0012081	CCTAGCACGATCCAAGCATTCTTC	TCACATAACACTCCGAGCCTCAG
GL50803_0011642	CGAAGCATAGAGGTTGTGGACAG	AGCGTTGACTTGGAAGAGTTGG
G6P-NAT	ACTGCCATCGTGCCCAAATCG	TGACCCAGGTCGGTGACTTCG
MDH	AAGCCCGTTCTCCGTGTTTGC	TCGTAGCCCAGCATGTCTCCAG
GL50803_0017165	AGGAGATGGACGAGCTGCTCAC	TGCGATCCACCCGAACATTGC
GL50803_003470	CGCCATGCTTCAGACGGATACC	GCGACCAGAGCACCGATTCAC
GL50803_008227	GTTGGTTGCGGAGAAGGAGACG	CAGACAGGAGGGCGGACAGG
GL50803_0087446	ACCCTGTCCTCTGCTGCACTAC	GCACAGTGAGCCCCAGAAACTC
GL50803_008559	GCTCTTGACCCTCGTCTTTGGC	ACCATTGCTGCCGCTGAAGTTG

Table S4. Candidate *Giardia* proteins interacting with GLVCP identified by LC-MS/MS

Accession	Gene	Mw(kDa)	Peptides	Sequence coverage [%]
E2RU97	GL50803_006430	28.576	9	35.5
A8B484	GL50803_007082	9.673	2	33.7
A0A644F9A2	GL50803_0016265	28.364	2	8.5
A8BFE3	GL50803_0011359	30.412	4	16
A8B6L8	GL50803_0014620	28.028	4	16.9
A8BT38	GL50803_0014329	21.752	4	18.9
A8B252	GL50803_005845	19.413	6	42.5
A0A644FB17	GL50803_007766	28.072	3	16.3
A8BNT0	GL50803_0017054	35.036	4	14.1
A0A644FBU1	GL50803_0016387	20.453	4	20.9
A8B1T3	GL50803_008462	15.35	2	19.3
A8BLL7	GL50803_0017244	25.383	1	4.9
A8BAV7	GL50803_0035341	776.876	1	0.2

Accession	Gene	Mw(kDa)	Peptides	Sequence coverage [%]
A8BUY7	GL50803_0016867	96.36	7	11
A0A644F3U0	GL50803_0013608	76.672	7	12.6
A8BV70	GL50803_0040817	41.586	1	2.7
A8BUF3	GL50803_008726	106.494	1	1.3
A8B4I3	GL50803_0016667	99.472	5	6.5
A0A644F3M5	GL50803_0086511	99.039	11	17.2
E2RTW6	GL50803_00112885	19.436	1	8.9
A8BCC2	GL50803_0013930	20.397	1	7.5
A8BSW7	GL50803_007789	21.765	6	49.2
A0A644F460	GL50803_0015574	80.807	1	1.5
A0A644F722	GL50803_0013350	44.685	1	3
E2RTP3	GL50803_003593	44.824	5	15.2
E2RU89	GL50803_003861	45.228	3	8.6
A8B3J7	GL50803_0093358	97.134	1	1.5
A8BLJ4	GL50803_009008	34.765	1	4.3
E2RTP4	GL50803_0011654	33.89	6	27.8
E2RTR2	GL50803_006226	104.85	1	1.4
A0A644F7T7	GL50803_005649	33.522	1	4.4
E2RTU4	GL50803_0017153	35.011	7	31.6
A0A644F164	GL50803_0015097	37.734	3	13.3
E2RTW5	GL50803_004026	47.871	1	3.4
A8BI13	GL50803_0014551	33.148	5	24.9
E2RTP6	GL50803_00103373	42.91	6	12.9
A8BVK6	GL50803_004239	11.494	2	19.4
A0A644FAV9	GL50803_0015832	54.939	2	5.7
A8BCA2	GL50803_0017174	212.735	1	0.7
A8BA58	GL50803_00101168	125.034	3	5.1
A8BVE9	GL50803_00103783	25.603	5	27.3

Accession	Gene	Mw(kDa)	Peptides	Sequence coverage [%]
A8BKY7	GL50803_00113622	164.12	5	5.1
A8BM19	GL50803_0011720	48.468	1	2.9
A8B5K6	GL50803_0014859	102.71	2	2.8
A0A644EZI6	GL50803_0015304	164.91	1	0.9
A0A644F070	GL50803_0015410	27.662	1	5.6
A0A644F271	GL50803_0016326	78.929	1	1.1
A8BZE2	GL50803_0017046	74.579	2	3.7
A8B820	GL50803_0017060	66.236	3	6.4
A8BPT2	GL50803_0017551	118.714	3	4.3
A8BLM5	GL50803_0024009	46.684	2	6.9
A8B595	GL50803_005188	57.133	1	2.9
A8BR70	GL50803_006081	132.437	1	1.4
A8BPT8	GL50803_0088369	92.039	4	8.7
A8BXV9	GL50803_009030	36.635	4	18.8
A8B5M0	GL50803_009720	112.847	1	1.2
A8B9H1	GL50803_0012139	75.884	13	23.1
A8B5N2	GL50803_00137718	77.939	1	1.4
A8B9U8	GL50803_0021423	122.832	3	3.3
E2RU36	GL50803_00112103	64.132	15	33.6
A8B9N2	GL50803_0010521	70.295	1	1.6
A0A644F3F8	GL50803_007953	39.506	2	9.6
A8BG41	GL50803_002098	48.437	1	3
A8BQW1	GL50803_0015048	73.379	2	3.7
A0A644F983	GL50803_00137716	179.269	25	20.8
A8B615	GL50803_13475	236.334	1	2.5
E2RTU7	GL50803_004812	30.877	29	79.8
A0A644F4P9	GL50803_0017121	72.662	11	19.2
E2RU99	GL50803_006184	39.065	2	7.9

Accession	Gene	Mw(kDa)	Peptides	Sequence coverage [%]
A8B3S4	GL50803_00103709	94.748	1	2
A8B2K1	GL50803_0016935	93.684	16	28.5
E2RTT2	GL50803_005333	17.275	1	7.2
A8BBV1	GL50803_007207	120.103	5	7.2
A8BFE9	GL50803_0011354	30.099	1	3.6
A8BB85	GL50803_0016453	33.879	9	34.2
A8BTG7	GL50803_0014019	33.056	1	3.3
A8BA54	GL50803_0016504	97.344	1	1.2
E2RU85	GL50803_006744	18.687	1	5.6
A8BKU9	GL50803_0016926	56.805	2	4.4
A8BQU9	GL50803_009808	45.109	4	10.8
A8BFG8	GL50803_0017483	37.253	2	9.1
A8BYL1	GL50803_0013584	43.636	4	13.5
A8B8S8	GL50803_0015499	48.834	8	21.3
A0A644F191	GL50803_0017412	81.443	1	2.4
A8BBR3	GL50803_0014921	54.844	5	14.3
A8BWS4	GL50803_0013372	55.267	12	24.4
A8B754	GL50803_0015218	83.8	8	11.3
A8BTT3	GL50803_0016013	59.93	2	4.8
A8BIM8	GL50803_007234	41.033	1	2.6
A8BRN9	GL50803_006171	17.36	1	7.3
A8BC51	GL50803_0011867	47.842	7	25.9
A0A644F7M3	GL50803_0017058	117.46	1	1
A8BQK6	GL50803_008639	51.869	2	5
A8BY03	GL50803_0091374	98.93	10	14.1
A8BPD9	GL50803_0010882	33.117	2	10.3
A8B3M2	GL50803_004507	99.351	2	3
A8BDK0	GL50803_0015446	38.445	1	3.8

Accession	Gene	Mw(kDa)	Peptides	Sequence coverage [%]
A8BFF0	GL50803_0017478	601.116	1	0.1
A8BCR6	GL50803_0088765	71.633	10	17.8
A8BHN6	GL50803_0021628	42.378	6	20.4
A0A644F7Y6	GL50803_0034684	74.354	2	4.6
A0A644FAT1	GL50803_0086676	30.874	12	52.6
A8BQG5	GL50803_0016200	41.297	2	8
E2RTV5	GL50803_0016424	29.702	6	35.3
A0A644EYR3	GL50803_0017332	178.552	1	0.8
E2RU90	GL50803_0016975	168.4	1	0.7
A0A644F4E8	GL50803_0014413	9.482	2	32.1
A8BII4	GL50803_0017195	18.926	1	6.2
A8BKM5	GL50803_00112210	13.115	1	7.3
E2RU04	GL50803_0014373	79.513	10	16.8
A8BM39	GL50803_0033218	83.864	2	5
A8BVK2	GL50803_004236	11.889	1	13.1
A8BL61	GL50803_009848	10.448	2	24.7
A8BIK9	GL50803_003256	45.266	2	8.4
A8BVR3	GL50803_00112304	49.105	15	40.7
A8BFR0	GL50803_0012102	45.217	5	15.7
A8BU59	GL50803_0017570	99.626	7	10.1
A0A644FAR5	GL50803_0016996	39.591	8	26.3
A8B8Z3	GL50803_0014606	26.814	2	13.4
A0A644F4F4	GL50803_0014583	19.378	3	25.3
A8B5M1	GL50803_009719	47.004	2	4.4
A8BBJ1	GL50803_0016125	118.854	9	11.4
A8B433	GL50803_006633	46.231	1	3.2
A8BGY8	GL50803_0024451	15.473	2	23
A8BSY0	GL50803_41512	74.044	12	24.6

Accession	Gene	Mw(kDa)	Peptides	Sequence coverage [%]
A8B2U2	GL50803_0011043	35.214	2	9.3
A8BDZ8	GL50803_0016653	103.116	3	4
A0A644F0H9	GL50803_009605	70.641	5	9.3
A8B9S8	GL50803_0017460	51.371	1	3.9
E2RTN7	GL50803_0017230	35.632	17	51.4
A8BGU6	GL50803_0011487	64.898	1	2.6
E2RU75	GL50803_0017090	20.734	3	20.4
E2RTY6	GL50803_009115	64.493	1	1.9
A8BRP6	GL50803_007188	115.621	1	1.4
A8BFF8	GL50803_0021942	49.734	12	31
A8BRQ2	GL50803_007195	98.321	1	1.8
A8BSP4	GL50803_006687	36.336	7	28.3
A8B731	GL50803_0013467	35.048	7	32.6
A8B8U9	GL50803_00104866	22.68	3	20.6
A8BS62	GL50803_0015869	25.421	4	22.1
A8B8P7	GL50803_007569	21.262	4	29.3
A8BYU1	GL50803_007532	71.537	2	3.7
A0A644F9H9	GL50803_0098054	80.772	12	19.6
A8BQB8	GL50803_0016412	94.798	2	4.5
A8BEW2	GL50803_009183	213.748	2	1.3
A8BN55	GL50803_0089117	319.906	1	0.7
A8BI78	GL50803_00121045	14.59	3	28.5
E2RU29	GL50803_00135231	16.32	1	4.8
A8BUJ9	GL50803_00135002	11.1	2	20.2
A8B9N7	GL50803_0010524	27.283	3	19.2
D3KHD6	GL50803_0014198	42.513	7	25.1
A8B9W3	GL50803_0091354	63.489	17	52.7
A8B9X5	GL50803_009148	22.258	7	49.5

Accession	Gene	Mw(kDa)	Peptides	Sequence coverage [%]
A8BM93	GL50803_0010422	24.26	4	22.6
A8BE31	GL50803_0016648	88.365	10	15.4
A8BWK6	GL50803_00101326	35.101	5	28.4
A8B313	GL50803_0014584	109.943	2	2.6
E2RTY0	GL50803_0011364	65.976	1	3.1
A0A644FBG0	GL50803_0016235	38.413	2	7.1
A8B2A9	GL50803_0016802	32.862	3	14.1
A8BME4	GL50803_0011311	41.542	1	3.5
A8BLC3	GL50803_00113553	71.522	3	7.1
A8BH19	GL50803_0015409	57.01	2	6
A8BH24	GL50803_0015411	78.006	3	6.8
A8B9M4	GL50803_0026199	53.054	1	3.5
A8B355	GL50803_005489	61.33	2	5.6
A8BJX2	GL50803_0086934	32.385	6	27.9
A0A644F026	GL50803_0061271	60.811	4	11.1
A8B4Z7	GL50803_00102101	114.615	6	10.5
A8B518	GL50803_00112846	120.516	7	10.6
A8B4Z2	GL50803_006262	121.029	1	0.7
A8BQA5	GL50803_006404	84.115	1	1.6
D3KI37	GL50803_0016869	189.44	2	1.6
A8BRM7	GL50803_004590	30.773	6	23.9
A8BGG4	GL50803_005795	85.45	2	3.7
A8B6Y0	GL50803_00113143	467.5	1	0.2
A0A644FBC3	GL50803_0021118	82.265	1	1.5
A8BEL5	GL50803_0016766	65.971	1	2.4
E2RU65	GL50803_003331	35.494	2	13.9
A8BER9	GL50803_0016343	100.584	6	6.7
A8B7Y8	GL50803_0010892	80.735	1	1.5

Accession	Gene	Mw(kDa)	Peptides	Sequence coverage [%]
A8BAZ8	GL50803_0010808	25.237	4	25.9
A8BS66	GL50803_0022850	41.096	5	13.3
E2RTN5	GL50803_008917	48.414	3	8.1
A8BA81	GL50803_0040067	54.963	1	2.3
A8BSQ6	GL50803_003158	22.543	7	46.2
A0A644EYY1	GL50803_0033769	50.296	8	21
A8BSH8	GL50803_0017150	18.583	5	51.2
A8BSH5	GL50803_0017151	19.454	1	7.7
A8BYV1	GL50803_007537	46.484	2	5.7
A8BKM6	GL50803_008528	56.851	1	1.7
A8BBK6	GL50803_006724	13.447	2	23
A8BMM8	GL50803_0016371	77.617	1	2.2
A8BBN0	GL50803_005359	69.051	3	5.4
A8BEI4	GL50803_0016768	354.616	1	0.3
A8BMG9	GL50803_0011301	16.793	2	15.2
A8BLA6	GL50803_0014135	43.121	4	10.6
A8BT62	GL50803_0023357	52.112	1	2.4
A8BBM0	GL50803_00114246	45.385	1	3.2
A8B4W6	GL50803_00112875	435.768	1	0.4
E2RTT6	GL50803_0010311	36.437	5	19
A8BY53	GL50803_008619	60.889	1	1.9
A8B686	GL50803_0016353	83.653	2	3.3
A8BC67	GL50803_0017163	18.044	1	7.1
A8BK50	GL50803_0010570	38.873	2	9
A8BYC4	GL50803_0016076	22.54	3	14.4
A8BAD0	GL50803_0017255	73.714	1	1.8
A8BGW7	GL50803_0014404	42.229	3	10.6
A8BQI1	GL50803_004197	39.316	4	15.7

Accession	Gene	Mw(kDa)	Peptides	Sequence coverage [%]
E2RU18	GL50803_0017254	73.93	1	2
A8BJ46	GL50803_008822	65.091	2	5.3
E2RTS6	GL50803_0011118	48.22	6	21.1
A8BTM8	GL50803_0016844	34.535	14	58.6
A8B9U2	GL50803_0032658	120.264	2	2.2
A8BKJ8	GL50803_005942	50.023	2	5.4
A8BN04	GL50803_0096570	175.436	1	0.6
A8BTM3	GL50803_16843	137.56	2	1.3
E2RTR8	GL50803_0029487	25.777	4	16.2
E2RTZ3	GL50803_009413	50.408	4	9.6
A8B3I4	GL50803_00103713	40.342	3	11.6
A8BTN1	GL50803_004852	48.873	2	6.2
A0A644FBQ4	GL50803_0091348	86.22	8	15.3
A8BUZ8	GL50803_0024603	233.448	1	0.6
A8B8G6	GL50803_0015871	53.298	2	5.1
A8BCS1	GL50803_007031	109.263	1	1.3
A0A644F037	GL50803_0061270	92.44	1	1.3
A8BDW4	GL50803_0016817	52.409	2	4.6
A8B6X3	GL50803_0010255	43.273	6	18.2
A8BQ62	GL50803_005810	14.5	2	17.6
A8B498	GL50803_0017143	60.525	3	7.6
A8BW44	GL50803_003206	70.738	3	6
A8BQ26	GL50803_009909	97.659	16	22.9
E2RTZ1	GL50803_00114609	138.929	13	13.5
A8B852	GL50803_0017063	131.803	15	15.4
E2RU48	GL50803_001695	23.571	3	25
E2RTZ2	GL50803_009558	23.107	3	17.9
A8B742	GL50803_0016636	24.595	2	9.3

Accession	Gene	Mw(kDa)	Peptides	Sequence coverage [%]
A8BTG5	GL50803_0093550	282.136	1	0.5
A8B2Z9	GL50803_00113788	6.46	1	21.4
A8B772	GL50803_007244	21.178	1	6.2
A0A644FC41	GL50803_0016044	14.404	2	20.2
A8B481	GL50803_0034093	8.589	1	18.2
A0A644FB74	GL50803_001345	24.591	9	48.4
A8BI95	GL50803_005593	19.736	2	15.6
A8B8F8	GL50803_0014938	19.572	5	34.1
A8BU75	GL50803_0011247	22.873	1	6.1
A8B4F8	GL50803_0014091	14.76	3	37.4
A8BE76	GL50803_0098056	18.681	1	7.3
A8B4T7	GL50803_0011950	20.162	1	7.3
A8BH61	GL50803_0016431	23.137	3	16.3
E2RTN4	GL50803_0016086	27.037	1	4.4
A8B2Q4	GL50803_0015520	18.257	2	15.7
A8BS37	GL50803_0010091	15.422	2	14.8
A8BMP6	GL50803_007870	16.023	3	22
A8B7P2	GL50803_0014869	22.697	1	6.3
A8BFT3	GL50803_0015046	15.584	1	8.9
A8BZ78	GL50803_0016310	17.129	3	22.8
A8BRZ3	GL50803_0016525	42.642	10	34.6
A8B5N7	GL50803_0014321	11.685	2	16.5
A8BMP3	GL50803_0016368	12.158	4	33
A8BMD9	GL50803_0014049	15.948	1	8.1
A8BMF7	GL50803_0036069	13.498	1	13.3
A8BKK5	GL50803_005947	13.889	2	20.3
A8B7H8	GL50803_0017547	34.988	6	25.6
E2RU47	GL50803_0017395	33.895	6	23.2

Accession	Gene	Mw(kDa)	Peptides	Sequence coverage [%]
A8BNT5	GL50803_0017056	20.855	2	11.9
A8BRY6	GL50803_0019436	27.058	10	40.9
A8BCP0	GL50803_0016588	11.953	3	27.9
A8BRX3	GL50803_0010919	15.384	2	17.9
A8BVU0	GL50803_0014827	18.064	1	13
A8BUG8	GL50803_0033862	14.354	1	8
A8BE02	GL50803_0016652	17.591	1	5.8
E2RU83	GL50803_007878	15.726	3	24.8
A8BBP3	GL50803_0015260	16.692	2	17.9
E2RU77	GL50803_0015228	14.748	2	19.2
A8BKC6	GL50803_004652	17.599	2	20.9
A8BZ58	GL50803_0015551	17.472	2	14.9
A0A644F964	GL50803_0060211	15.435	3	25.9
A8BMZ1	GL50803_008118	26.724	8	37.2
A8B4G0	GL50803_006022	14.051	1	11.9
A8B593	GL50803_0010367	14.844	2	12.9
A0A644F5K9	GL50803_0010780	9.133	1	16
A8B4F3	GL50803_003570	7.346	2	28.1
A8B8Z2	GL50803_007999	24.738	3	12.9
A8BZD3	GL50803_0012981	21.025	1	7.9
A8BKS8	GL50803_004547	21.653	6	35.4
A8BL74	GL50803_009861	42.712	8	23.4
E2RTX5	GL50803_003036	14.801	1	6.9
A8BBV4	GL50803_007204	18.593	1	6.9
A8BGC8	GL50803_009825	51.418	2	5.1
E2RUA1	GL50803_004410	29.828	16	45.5
A8B7R2	GL50803_0014874	21.453	1	8.5
A0A644EYU5	GL50803_0017330	18.716	1	8.2

Accession	Gene	Mw(kDa)	Peptides	Sequence coverage [%]
A8BR91	GL50803_5744	54.093	1	1.2
A0A644F8I9	GL50803_0060210	13.191	1	5.9
A8BYY5	GL50803_007439	71.769	1	2.3
D3KH10	GL50803_0015445	32.248	3	11.2
A8B747	GL50803_0015214	35.474	4	16.2
A0A644FAU7	GL50803_005010	35.446	1	4.9
A8BMB1	GL50803_004043	118.901	1	0.7
A8BHP4	GL50803_0095908	423.538	2	0.5
A0A644F6U2	GL50803_0012224	33.878	8	31.5
A8BHT5	GL50803_003920	81.529	1	2.3
A8BNT2	GL50803_0017055	62.272	1	2.2
A8BRD5	GL50803_0017375	40.588	2	9.9
A8BX22	GL50803_0027310	65.361	1	1.9
E2RTR0	GL50803_0091919	59.281	4	10
E2RTV1	GL50803_0011992	61.193	1	2.5
A8BBI5	GL50803_0016124	64.753	1	3.7
A8BD81	GL50803_006812	14.185	1	8.8
A8B2K9	GL50803_0016934	155.223	1	0.7
D3KI75	GL50803_0087577	33.311	7	32.8
E2RU27	GL50803_009827	33.869	3	17.8
A8BCK8	GL50803_27745	4.671	1	22
A8BVD3	GL50803_009704	77.564	3	5
A8BZ22	GL50803_0013561	24.759	1	6.4
A8BBN6	GL50803_006242	17.368	2	14.6
A8BU68	GL50803_0017571	116.396	13	15.3
A8B9P6	GL50803_0032375	156.25	4	3.9
A0A644F2V1	GL50803_0015427	25.296	4	24.4
A8BA49	GL50803_0016507	98.639	1	2.3

Accession	Gene	Mw(kDa)	Peptides	Sequence coverage [%]
A8BPC0	GL50803_00103676	50.552	18	53.1
A8BEI6	GL50803_00101291	50.05	18	49.2
A0A644F2V5	GL50803_0023300	8.936	2	35.5
A8BJ08	GL50803_007110	9.291	4	57.3
A8BAA3	GL50803_0094463	53.39	7	18.8
A8B8W8	GL50803_0010025	27.232	3	13.7
D3KFZ2	GL50803_0011548	28.856	1	3.1
A8BKB9	GL50803_0015139	19.433	2	17.3
A8B9R3	GL50803_0015240	46.451	1	1.9
A8BST0	GL50803_0015918	24.79	9	49.6
A8BVS0	GL50803_0016861	33.656	2	12.4
A0A644F6S5	GL50803_0029796	40.624	5	17.5
A8B7W2	GL50803_003934	22.366	3	20.8
A8BKU5	GL50803_005890	12.036	1	9.4
D3KHC7	GL50803_006558	35.895	3	11.1
A8BK27	GL50803_007825	22.676	2	17.1
A8BU37	GL50803_20889	14.279	1	4.8
E2RU95	GL50803_009779	34.283	4	12.6
E2RTS0	GL50803_006563	23.2	1	6.2
A8BIZ7	GL50803_0016549	77.208	1	2.5
A8BYJ2	GL50803_008217	66.044	2	4.8
A8BRI9	GL50803_0013272	40.411	2	8.4
A8BPV3	GL50803_0012216	54.747	2	4.6
A8BHQ3	GL50803_007323	22.905	2	13.6
A0A644F867	GL50803_00100864	54.676	2	6.5
E2RU40	GL50803_00101906	49.725	1	3.5
A8BTY2	GL50803_003582	40.313	1	3
A8B6Z3	GL50803_0010822	38.531	2	6.4

Accession	Gene	Mw(kDa)	Peptides	Sequence coverage [%]
A8BV56	GL50803_0033762	33.106	2	6.6
A8BM82	GL50803_0010429	20.852	3	18.9
A8BK79	GL50803_0017327	49.672	5	17.8

Table S6. The top10 interaction sites between UCP and GLVCP predicted by AlphaFold 3

UCP	GLVCP	Distance	Specific interactions	Surface complementarity
Asn 93	Ser 544	2.1	1x hb to Ser 544	0.86
Arg 156	Phe 155	2.4	1x hb to Phe 155	0.86
Val 192	Asn 487	2.2	1x hb to Asn 487	0.85
Gln 419	Ala 667	2.3	1x hb to Ala 667	0.84
Gly 472	Tyr 147	1.9	1x hb to Tyr 147	0.83
Ala 431	Val 538	2.1	1x hb to Val 538	0.81
Val 417	Leu 651	2.2	1x hb to Leu 651	0.8
Lys 177	Leu 128	2	1x hb to Leu 128	0.78
Asp 155	Tyr 157	1.6	1x hb to Tyr 157	0.77

Notes: Distance: The distance between two protein amino acids. **Specific Interactions:** Whether two protein amino acids form a binding bond and the specific information about the bond. **Surface Complementarity:** Surface complementarity, which describes the degree of matching between the surfaces of two or more molecules. This value ranges from 0 to 1, with values closer to 1 indicating a higher likelihood of interaction between the two amino acids.
